# Stimulation of Gross Chromosomal Rearrangements by the Human CEB1 and CEB25 Minisatellites in *Saccharomyces cerevisiae* Depends on G-Quadruplexes or Cdc13

**DOI:** 10.1371/journal.pgen.1003033

**Published:** 2012-11-01

**Authors:** Aurèle Piazza, Alexandre Serero, Jean-Baptiste Boulé, Patricia Legoix-Né, Judith Lopes, Alain Nicolas

**Affiliations:** 1 Recombinaison et Instabilité Génétique, Institut Curie Centre de Recherche, CNRS UMR3244, Université Pierre et Marie Curie, Paris, France; 2 NGS Platform, Institut Curie, Paris, France; University of Washington, United States of America

## Abstract

Genomes contain tandem repeats that are at risk of internal rearrangements and a threat to genome integrity. Here, we investigated the behavior of the human subtelomeric minisatellites HRAS1, CEB1, and CEB25 in *Saccharomyces cerevisiae*. In mitotically growing wild-type cells, these GC–rich tandem arrays stimulate the rate of gross chromosomal rearrangements (GCR) by 20, 1,620, and 276,000-fold, respectively. In the absence of the Pif1 helicase, known to inhibit GCR by telomere addition and to unwind G-quadruplexes, the GCR rate is further increased in the presence of CEB1, by 385-fold compared to the *pif1Δ* control strain. The behavior of CEB1 is strongly dependent on its capacity to form G-quadruplexes, since the treatment of WT cells with the Phen-DC_3_ G-quadruplex ligand has a 52-fold stimulating effect while the mutation of the G-quadruplex-forming motif reduced the GCR rate 30-fold in WT and 100-fold in *pif1Δ* cells. The GCR events are telomere additions within CEB1. Differently, the extreme stimulation of CEB25 GCR depends on its affinity for Cdc13, which binds the TG-rich ssDNA telomere overhang. This property confers a biased orientation-dependent behavior to CEB25, while CEB1 and HRAS1 increase GCR similarly in either orientation. Furthermore, we analyzed the minisatellites‚ distribution in the human genome and discuss their potential role to trigger subtelomeric rearrangements.

## Author Summary

All genomes contain particular DNA sequences that are prone to break and rearrange. They can be lost or rescued at the expense of sequence variations and complex rearrangements. Using a sensitive yeast model system, we examined the fragility of the HRAS1, CEB1, and CEB25 GC-rich human minisatellites (tandem repetition of motifs from 10 to 100 bp long). We observed that they all stimulate Gross Chromosomal Rearrangements but to very different extents, both in wild type and in cells deficient for the Pif1 helicase. Several intrinsic sequence features can account for these differences: the total number of repeats, the ability to form G-quadruplex secondary structures, or the ability to bind with high affinity the telomerase cofactor Cdc13. The orientation on the chromosome dictates the type of GCR (telomere addition versus other structural rearrangements) while not affecting the GCR rate in most cases. Being enriched in the subtelomeric regions of the human chromosomes, this class of GC–rich minisatellite has the potential to trigger a large variety of human genome rearrangements.

## Introduction

Some chromosomal regions are more prone to rearrangement than others and thus are the source of genetic diseases and cancer. Among “at risk” sequences, tandem repeats like microsatellites and minisatellites that differ by the length of their repeat unit (1–10 nt and 10–100 nt, respectively) are prone to changes in repeat number (expansion and contraction of the array) [1]. Mechanistically, this instability can be explained by the propensity of the motifs to misalign during template-directed repair of endogenous lesions, occurring stochastically or promoted by the nucleotide sequence themselves, which, for example, can perturb replication. Consistently, their instability is exacerbated by defects of replication proteins (like Rad27 or Polδ) that ubiquitously affect genome integrity [2]–[7].

Intrinsic features of repeated sequences also play a role in the formation of rearrangements [1]. Microsatellite instability caused by hairpin formation during replication has been well documented [8] but less is known about minisatellite instability. Sequence composition and its ability to interact with endogenous factors and/or to adopt secondary structures can be invoked. Among these are G-quadruplexes. They are four-stranded structures that some G-rich nucleic acids form spontaneously in physiological salt and pH conditions *in vitro* [9]. A growing body of evidence implicates these structures in several biological processes, like directed genome rearrangements [10], [11], telomere capping [12], [13], and control of gene expression at the transcriptional and post-transcriptional levels [14], [15]. Recently, we showed that the GC-rich human minisatellite CEB1 forms G-quadruplexes *in vitro* and demonstrated that Pif1, a conserved 5′-3′ helicase, unwinds these G-quadruplexes [16]. In *Saccharomyces cerevisiae*, Pif1 prevents the formation of G-quadruplex-dependent CEB1 internal rearrangements during leading-strand replication and, consistently, the treatment of WT cells with the potent G-quadruplex binder Phen-DC_3_ mimicks the absence of Pif1 [16], [17], [18].

A different but perhaps related feature of the human GC-rich minisatellites with respect to genome stability is their clustering in the chromosomal subtelomeric regions [19], [20] that are subjected to pathological terminal truncations [21]–[24]. The genomic factors involved in the highly dynamic behavior of terminal regions being poorly identified, here we examined the fragility of the subtelomeric human minisatellites HRAS1 [25], CEB1 [26] and CEB25 [27] and the role of their specific sequence features in the induction of Gross Chromosomal Rearrangements (GCR) in *S. cerevisiae*. To this end, we employed the GCR assay developed by R. Kolodner and colleagues [28] that measures the rate of the yeast chromosome V terminal deletion. We showed that the three minisatellites and sequence variants stimulated the formation of GCR in WT cells to different extents depending on several factors: the number of motifs in the tandem array, the ability to form G-quadruplexes, the presence of Cdc13 binding sites, their orientation which yields different type of rearrangements, and/or the activity of Pif1 and of the homologous recombination pathway. Altogether, these results point to GC-rich minisatellites as major at-risk regions of the genome not only for changes in repeat number but also for their propensity to generate structural variants.

## Results

### Experimental system

**Figure 1:**
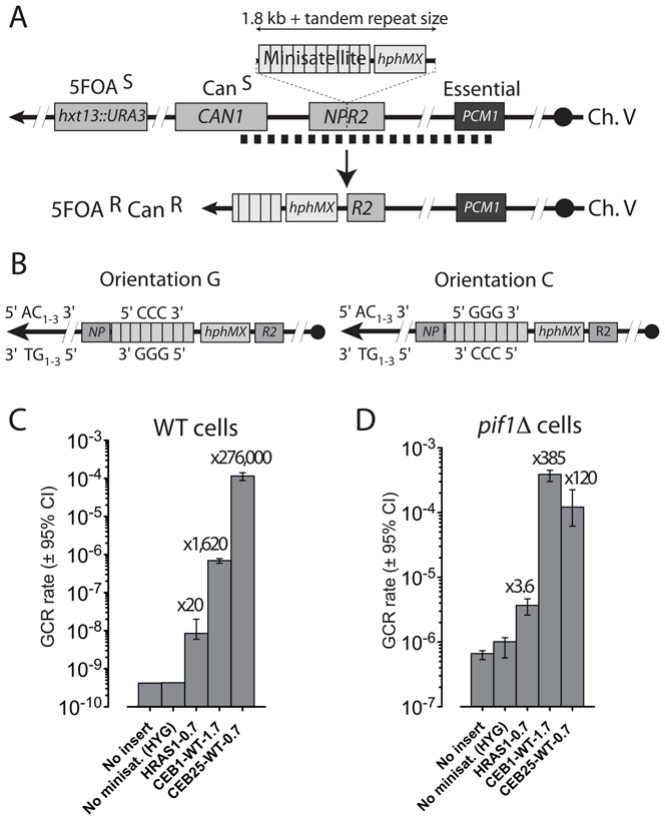
GC–rich minisatellites induce the formation of GCR in WT and *pif1Δ* cells. (A) Schematic representation of the GCR assay on the *S. cerevisiae* chromosome V. *URA3* has been inserted at the *HXT13* locus (*hxt13::URA3*) [28]. The *CAN1* and *URA3* genes confer sensitivity to canavanine (Can) and 5-fluoroorotic acid (5FOA), respectively. Plating cells on media containing both Can and 5FOA allows detecting the simultaneous loss of both markers, occurring upon genomic rearrangements (GCR) in the non-essential region between the first essential gene *PCM1* and *CAN1* (≈13 kb, dotted line) [28]. The rate of GCR is determined by fluctuation analysis of 5FOA/Can-resistant colonies occurrence (see Materials and Methods). A cassette containing the *hphMX* gene and the minisatellite of interest is inserted in the closest centromere-proximal position to the *CAN1* and *URA3* genes, at the *NPR2* locus. The example of a telomere addition in the tandem repeat associated with the loss of the distal part of the chromosome V is shown. (B) Schematic representation of the two orientations in which CEB1 has been inserted relatively to the distal telomere. (C) GCR rates in WT strains bearing no insert (RDKY3615), no minisatellite (control *npr2::hphMX* strain, ORT6531), HRAS1-0.7 (ORT7182), CEB1-WT-1.7 (ORT6542-6), or CEB25-WT-0.7 (ORT6558) in the orientation G. The fold increase over the “no minisatellite” control strain is indicated. (D) GCR rates in *pif1Δ* cells bearing either no insert (RDKY4399), no minisatellite (control *npr2::hphMX* strain, ORT6568), HRAS1-0.7 (ORT7122), CEB1-WT-1.7 (ORT6543-1), or CEB25-WT-0.7 (ORT6559-5) in the orientation G. Other legends as in (C).

To study the behavior of human GC-rich minisatellites in the formation of GCR, we employed the genetic assay developed by Chen and Kolodner [28]. In this sensitive assay, the left arm of chromosome V was engineered to measure the rate of the simultaneous loss of the *CAN1* and *URA3* markers located in the terminal non-essential part of the chromosome V. Cells that undergo a GCR event that results in the simultaneous loss of URA3 and CAN1 are recovered on media containing canavanine and 5-fluoro-orotic acid (5-FOA). Fluctuation analysis of the number of growing colonies provide a very sensitive GCR assay (see Materials and Methods), ranging over several order of magnitude since in WT cells, the GCR rate is approximately 10^−10^ events per generation [28]. We inserted the minisatellites centromere-proximal to *CAN1* within the non-essential *NPR2* locus, together with the Hygromycin resistance gene (*hphMX*) (Figure 1A). Importantly, the HYG^R^ cassette has a GC-content of 58%, does not share homology with the yeast genome, and is devoid of potential G-quadruplex-forming sequences or Cdc13 binding sites. Hereafter, to compare strains with similar size inserts, the hphMX construct constitutes our “no minisatellite” control strain.

Altogether, we examined three subtelomeric GC-rich human minisatellites: CEB1 [26], CEB25 [19], and the minisatellite located in the promoter of the HRAS1 gene [25]. They are tandem arrays with motif lengths of 39, 52, and 28 nt, respectively. The sequence of the consensus motif and additional features of these minisatellites are indicated in Table 1. Furthermore, it is known that the CEB1 and CEB25, but not the HRAS1 motifs, can form stable G-quadruplex structures *in vitro* [16], [27]. All three minisatellites were inserted in both chromosomal orientations at the same locus. In the orientation ‘“G”, the G-rich strands of CEB1 is on the same strand as the G-rich 3′ ssDNA overhang of the chromosome V left-arm telomere (distance is approximately 45 Kb), while in the orientation “C”, the C-rich strand is on the same strand as the G-rich 3′ overhang (Figure 1B). All the rates measured throughout this study are reported in Table S3. Hereafter, unless otherwise stated, the inserts we refer to are in the “G” orientation.

**Table 1:** Human minisatellites used in this study and their sequence composition properties.

Minisatellite	Location	Distance to Telomere	Motif size	% GC	GC-bias	TG/GG/GT-bias	G-rich strand motif sequence (5′-3′)
CEB1	2q37.3	0.4 Mb	39 bp	77%	77%	87%	**GGGGGG**A**GGG**A**GGG**TGGCCTGCGGAGGTCCCT**GGG**CTGA
CEB25	10q26.3	1.4 Mb	52 bp	56%	100%	100%	AA**GGG**T**GGG**TGTAAGTGT**GGG**T**GGG**TGTGAGTGT**GGG**TGTGGAGGTAGATGT
HRAS1	11p15.5	0.5 Mb	28 bp	68%	74%	80%	CCCCTGGAGAGAAGGGGGAGTGTGGCGT

Guanines potentially involved in the formation of G-quadruplexes are shown in bold.

### The minisatellites increase the GCR rate in WT cells to very different extents

First, we examined GCR rates in the absence of minisatellite inserts. Previous studies provided an estimated rate of 3.5.10^−10^ events/generation for WT cells [28]. Consistently, the control strain (*npr2::hphMX*) exhibits the same GCR rate as the parental (*NPR2^+^*) RDKY3615 haploid strain (4.3×10^−10^ vs. 4.2×10^−10^ events/generation, respectively). Thus, adding approximately 1.8 Kb of a non-repeated GC-rich DNA to the 13 kb region permissive for rearrangements (located between *CAN1* and the first centromere-proximal essential gen, *PCM1*) has no detectable effect. Then, we measured the consequence of the insertion of the CEB1-WT-1.7 allele containing 43 motifs [16], CEB25-WT-0.7 (13 motifs) and HRAS1-0.7 (26 motifs). Compared to the control strain (*hphMX*), these minisatellites strongly but differentially increased the GCR rate in WT cells: 20-fold for HRAS1 (8.48×10^−9^ events/generation), 1,620-fold for CEB1 (6.97×10^−7^ events/generation) and 276,000-fold for CEB25 (1.16×10^−4^ events/generation) (Figure 1C).

### Pif1 differentially suppress minisatellite-induced GCR formation

Pif1 is a conserved 5′-3′ helicase that suppresses GCR events by telomere healing [29], [30] through direct removal of the telomerase from DNA ends [31]. Pif1 is also involved in G-quadruplex unwinding [16]. We constructed *pif1Δ* cells carrying the minisatellites. Consistent with previous findings [29], [32], in the “no-insert” and in our control insert strain, the GCR rates are increased approximately 1500–2250-fold (6.63×10^−7^ and 1.01×10^−6^ events/generation, respectively) in the *pif1Δ* strain compared to WT. The presence of the minisatellites had various quantitative effects. Compared to the control *pif1Δ* strains, HRAS1, CEB1 and CEB25 stimulated the GCR rate 3.6-fold (3.68×10^−6^ events/generation), 385-fold (3.89×10^−4^ events/generation) and 120-fold (1.21×10^−4^ events/generation), respectively (Figure 1D). If we now compare the WT and the *pif1Δ* cells carrying the same minisatellite, the absence of Pif1 increases the GCR rate of HRAS1 and CEB1 approximately 500- and 558-fold, but has no effect on CEB25. This insensitivity to Pif1 reflects the already high rate of GCR induced by CEB25 in WT cells. The heterogeneous behavior of this set of minisatellites suggests that specific sequence features modulate their propensity to trigger GCR, in both WT and *pif1Δ* cells.

### The G-quadruplex-forming sequences of CEB1 stimulate the formation of GCR

**Figure 2:**
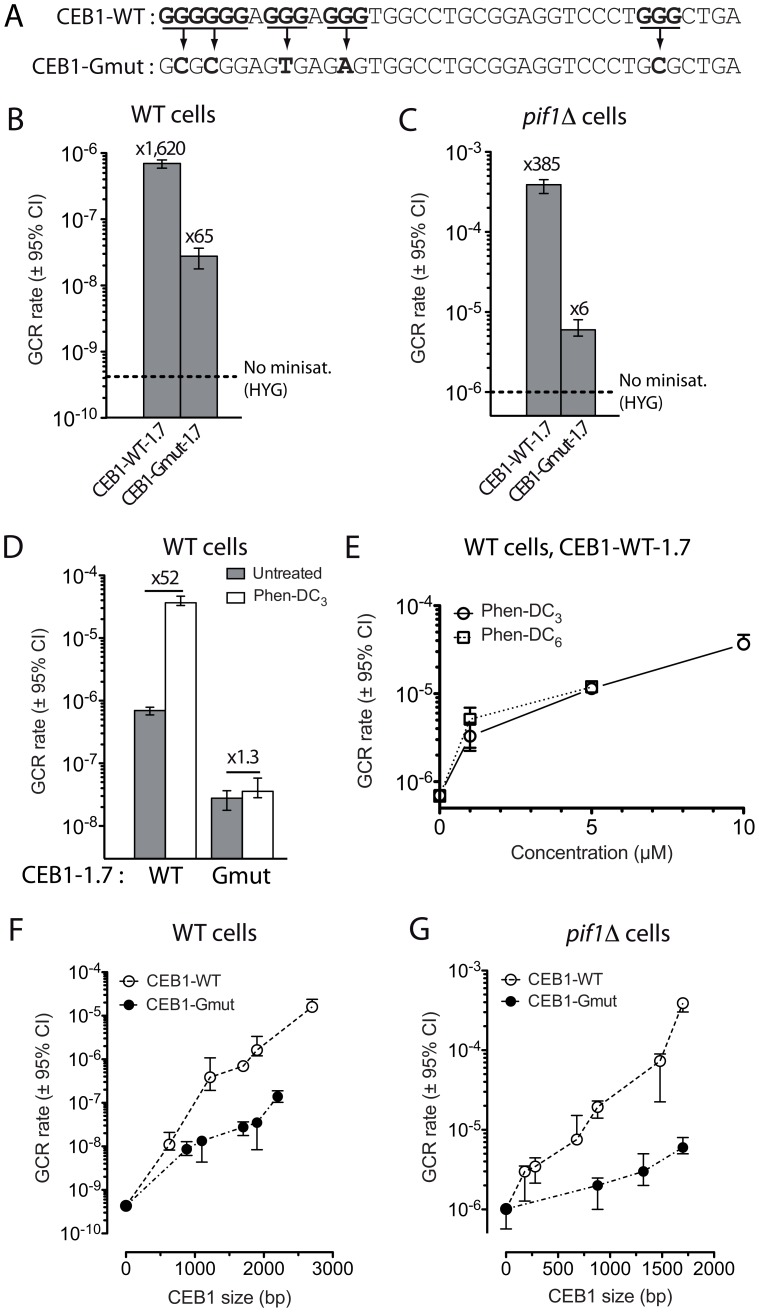
Size-dependent minisatellite fragility is aggravated by its ability to form G-quadruplexes, especially in G-quadruplex-stabilizing conditions. (A) Sequence of the CEB1-WT and CEB1-Gmut motifs used in this study [16], [18]. G-tracts potentially involved in the G-quadruplex formation are in bold and underlined. Single nucleotide mutations are depicted in bold. (B) GCR rates in WT cells bearing either CEB1-WT-1.7 (ORT6542-6) or CEB1-Gmut-1.7 (ORT6550-2). The dotted line indicates the GCR rate in the “no minisatellite” WT control strain, in which *hphMX* alone has been inserted at *NPR2* (ORT6531)(Figure 1C). (C) GCR rates in *pif1Δ* cells bearing either CEB1-WT-1.7 (ORT6543-1) or CEB1-Gmut-1.7 (ORT6551-1). The dotted line indicates the GCR rate in the “no minisatellite” *pif1Δ* control strain, in which *hphMX* alone has been inserted at *NPR2* (ORT6568)(Figure 1D). (D) GCR rates in untreated (grey) and Phen-DC_3_-treated (white) WT cells bearing either CEB1-WT-1.7 (ORT6542-6) or CEB1-Gmut-1.7 (ORT6550-2). The increase of the GCR rate upon treatment with Phen-DC_3_ is indicated. (E) GCR rates in the WT strain bearing CEB1-WT-1.7 in the orientation G (ORT6542-6) treated with 1, 5, and 10 µM Phen-DC_3_ (white circles) and 1 and 5 µM Phen-DC_6_ (with squares). GCR rates in WT (F) and *pif1Δ* (G) cells as a function of the size of CEB1-WT (open) and CEB1-Gmut (black). For each genotype, the point at 0 is the “no minisatellite” control strain (ORT6531 and ORT6568, respectively).

The CEB1 motif forms G-quadruplexes that are efficiently unwound by Pif1 *in vitro* [16], [18]. To determine the role of the G-quadruplex forming sequences of CEB1 on GCR rate, we first examined the behavior of the CEB1-Gmut-1.7 array which does not form G-quadruplex (Figure 2A) [16]. In the WT strain background, the insertion of CEB1-Gmut-1.7 yields a GCR rate of 2.06×10^−8^ events/generation. This is 65-fold higher than in the control strain but 30-fold lower than in the CEB1-WT-1.7 cells carrying the same number of G quadruplex forming motifs (Figure 2B). These results indicate that the effect of CEB1 on GCR rate is both G-quadruplex-independent and –dependent. Similarly, we examined the behavior of the CEB1-Gmut-1.7 allele in *pif1Δ* cells. The GCR rate was stimulated 6-fold (6.32×10^−6^ events/generation) compared to the control *pif1Δ* strain, but was 62-fold lower than in the CEB1-WT-1.7 cells (Figure 2C). This level is similar to the GCR rate induction observed with the HRAS1-0.7 minisatellite also devoid of G-quadruplex-forming sequence. We conclude that, in both WT and *pif1Δ* cells, the induction of GCR by CEB1 strongly depends on its potential to form G-quadruplexes.

To confirm the stimulating role of G-quadruplex, we compared the rate of GCR in cells treated or not with the G-quadruplex-stabilizing ligand Phen-DC_3_ [33]. The treatment of WT cells bearing CEB1-WT-1.7 with 10 µM Phen-DC_3_ yielded a GCR rate of 3.65×10^−5^ events/generation, 52-fold higher than in the untreated cells (Figure 2D). We verified that this induction was not due to a better growth rate of cells having performed a GCR in the presence of the ligand (Figure S1). In contrast, Phen-DC_3_ failed to increase the GCR rate in CEB1-Gmut-1.7 cells (3.56×10^−8^ events/generation) (Figure 2D). We also assayed concentration effects and treatment with Phen-DC_6_, a compound related to Phen-DC_3_ [33]. Clearly, the extent of GCR rate induction in WT cells carrying the CEB1-WT-1.7 minisatellite was stimulated by both ligands and is dependent on their concentration (Figure 2E).

Finally, since our previous studies examined G-quadruplex-dependent expansion/contraction of CEB1 in different chromosomal locations [16]–[18], we determined the frequencies of CEB1 expansion/contraction in this chromosome V location. As previously observed on Chr. III and VIII, the CEB1-WT-1.7 array was rather stable in WT cells (3/192 rearrangements) and became frequently rearranged upon treatment with Phen-DC_3_ (39/192, p-value vs. untreated = 8.8e^−10^) or *PIF1* deletion (16/192, p-value vs. WT = 3.55e^−3^) (Table 2). This depends on the presence of the G-quadruplex-forming sequences, since the CEB1-Gmut-1.7 allele remained stable in the above conditions (Table 2).

**Table 2:** Internal rearrangement frequencies of CEB1 in WT cells treated or not with 10 µM Phen-DC_3_, and in *pif1Δ* cells.

Minisatellite	Genotype	Untreated	Phen-DC_3_ 10 µM
**CEB1-WT-1.7**	WT	3/192 (1.6%)	39/192 (20.3%)*
	*pif1Δ*	16/192 (8.3%)°	ND
**CEB1-Gmut-1.7**	WT	1/192 (0.5%)	0/96
	*pif1Δ*	0/192	ND

* p-value vs. Control<0.05.

° p-value vs. WT<0.05.

We conclude that the impairment of the G-quadruplex unwinding capability of the cell, either by adding G-quadruplex-stabilizing ligands in WT cells or by deleting *PIF1*, stimulates the propensity of the G-quadruplex-prone CEB1 minisatellite to undergo a high level of expansion/contraction and to a lesser extent GCRs.

### CEB1 induces GCR in a size-dependent manner

Next, we examined the relationship linking the total number of motifs in the CEB1 array and the GCR rate, both in WT and *pif1Δ* cells. We observed that the rate of GCR in WT cells was positively correlated to the number of repeats (p-value = 2.8×10^−3^, Spearman's correlation test)(Figure 2F), with rates ranging from 1.1×10^−8^ events/generation for the allele of 0.66 kb (17 motifs) to 1.59×10^−5^ events/generation (37,000-fold higher) for the longest allele of 2.7 kb (≈70 motifs). The straight slope in logarithmic scale suggests that the relationship linking the motif number and the GCR rate is roughly exponential. Similarly, CEB1-Gmut also induces the formation of GCR in a size-dependent manner (p-value = 2.8×10^−3^) (Figure 2F), but with a lower slope: an allele of 1.9 kb (≈49 motifs) induced a GCR rate only 4-fold higher than a 0.9 kb allele (23 motifs)(3.52×10^−8^ and 8.64×10^−9^ events/generation, respectively). In the absence of Pif1, the GCR rates also increased exponentially with the number of CEB1-WT repeats (p-value = 3.97×10^−4^)(Figure 2G). Hence, we conclude that the number of repetition of the minisatellite motif is an aggravating factor of the fragility of these sequences, being steeper with the G-quadruplex-forming ones.

### The GCR induced by CEB1 are mainly CEB1 truncation healed by telomere addition

**Figure 3:**
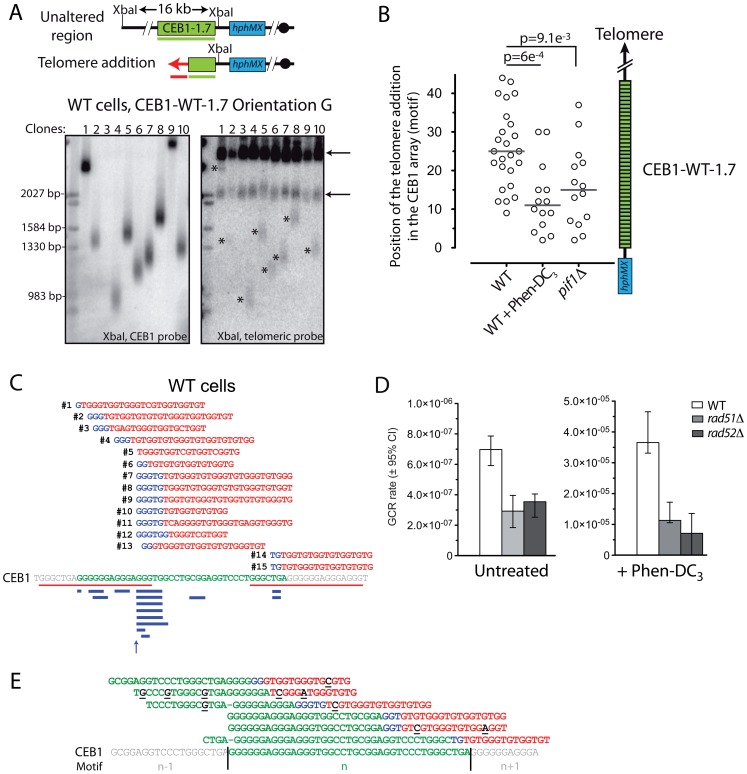
GCR are mainly telomere additions in CEB1. (A) The top panel schematically represents the genomic region surrounding CEB1 with the XbaI restriction site and the CEB1 (green) and TG_1–3_ (red) probes used to study rearrangements of the region. The size of the unaltered region upon digestion is indicated. Upon telomere addition, digestion with XbaI is expected to produce a fragment that can be hybridized by both the CEB1 and the telomeric (TG_1–3_) probe. The bottom panels show the rearrangements present in independent 5FOA/Can-resistant colonies obtained from WT cells bearing CEB1-WT-1.7 in the orientation G (ORT6542-6). Stars indicate smears hybridizing both the CEB1 and the telomeric probes (Orientation G lanes 1,2,4,5,6,7,8,10). Arrows indicate endogenous telomeres. Ambiguities remained for CEB1 smears co-migrating with endogenous telomeres (clone 9). One lane shows no CEB1 signal (clone 3). (B) Distribution of the telomere addition positions within CEB1-WT-1.7 estimated from the mean molecular weight determined by Southern blot (see Materials and Methods) in untreated and Phen-DC_3_-treated WT cells (ORT6542-6), and *pif1Δ* cells (ORT6543-1). Grey bars show the median of the distributions. Distributions were compared using a non-parametric test (Mann-Whitney-Wilcoxon). (C) Analysis of the CEB1-telomere junctions in WT cells (ORT6542-6). Each CEB1-telomere junctions have been obtained from 15 independent 5FOA/Can-resistant colonies. The sequences are oriented 5′-3′. The template CEB1 motif is shown in green, and the flanking motifs (n−1 and n+1) are shown in grey. The G-quadruplex forming motifs are underlined in red. Nucleotides shared by both the CEB1 and the telomeric sequences at the junction are in blue. The telomeric sequence is in red. The CEB1 sequence above the junctions is identical to the CEB1 reference, and is not shown for each molecule. A schematic representation of the length of shared nucleotides between the CEB1 and the telomeric sequences is shown in blue below the reference CEB1 motif. An arrow indicates the junction in which no nucleotide is shared. (D) GCR rates in WT (ORT6542-6), *rad51Δ* (ORT7189), and *rad52Δ* (ORT7310-2) cells bearing CEB1-WT-1.7 upon treatment (right) or not (left) with Phen-DC_3_ at 10 µM. The scale is linear. (E) Analysis of the single nucleotide polymorphisms (SNPs) around the CEB1-telomere junctions covered by at least 3 different reads. SNPs present in 100% of the reads are shown in black and underlined. Other legends as in (C).

**Figure 4:**
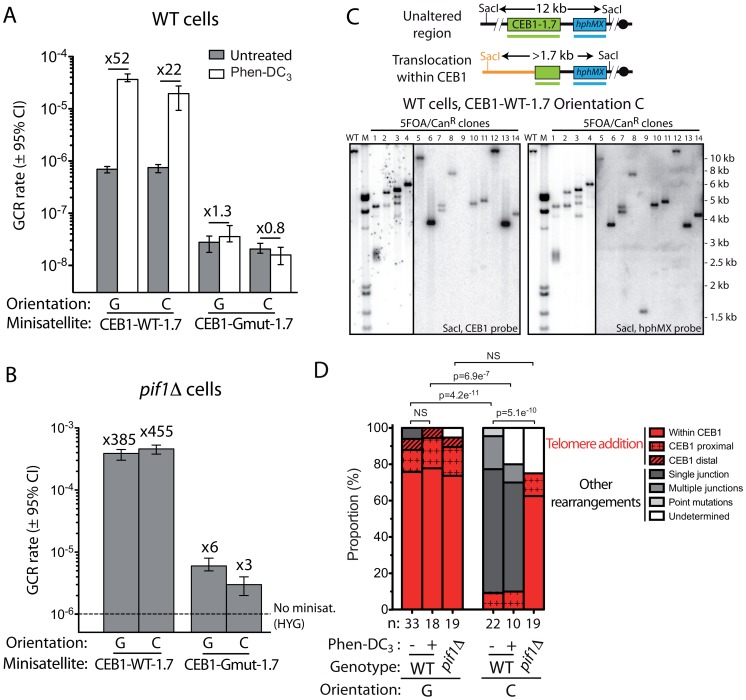
The nature of the GCR, but not the GCR rate, depends on the orientation of CEB1 in WT cells. (A) GCR rates in untreated (grey) and Phen-DC_3_-treated (white) WT cells bearing CEB1-WT-1.7 in the orientation G (ORT6542-6) and C (ORT6591-1), and CEB1-Gmut-1.7 in the orientation G (ORT6550-2) and C (ORT6548). The fold increases of the GCR rate upon treatment with Phen-DC_3_ is indicated. (B) GCR rates in *pif1Δ* cells bearing CEB1-WT-1.7 in the orientation G (ORT6543-1) and C (ORT7153-9), and CEB1-Gmut-1.7 in the orientation G (ORT6551-1) and C (ORT6549). The dotted line indicates the GCR rate in the “no minisatellite” control *pif1Δ* strain (ORT6568) (see Figure 1D). Other legends as in Figure 1D. (C) The top panel schematically represents the genomic region surrounding CEB1 with the *Sac*I restriction site and CEB1 (green) and *hphMX* (blue) probes used to study rearrangements of the region. The size of the unaltered region upon digestion is indicated. The example of a translocation within CEB1 is shown, and is expected to produce a fragment longer than 1.7 kb. The bottom panels show the rearrangements present in 14 5FOA/Can-resistant clones obtained from independent cultures of WT cells bearing CEB1-WT-1.7 in the orientation C, and in the parental 5FOA/Can-sensitive strain (WT, ORT6591-1). In most lanes the bands hybridized both the CEB1 and the *hphMX* probes, except in clone 9 in which CEB1 has been lost. M = Size marker. (D) Nature of the GCR determined by Southern blot analysis of independent 5FOA/Can-resistant colonies derived of cells bearing CEB1-WT-1.7 in the orientations G or C, in WT cells treated or not with Phen-DC_3_ 10 µM, or in *pif1Δ* cells. The results are presented as a percentage of the total number (n) of 5FOA/Can-resistant colonies analyzed. Telomere additions are shown in red, and their locations relative to CEB1 are indicated by different motifs: within CEB1 (no motif), in proximal (cross), or in distal (lines) position to CEB1. Other rearrangements that appear as discrete bands on the Southern blots are shown in grey: single junction (only one band) and multiple junctions (more than one band) are shown in dark grey and grey, respectively. The strain point mutated for *URA3* and *CAN1* (point mutations) is shown in light grey. In some instances, colonies have lost both CEB1 and *hphMX* and the junction has not been determined (Undetermined, white): this may correspond to telomere additions or other rearrangements in the 8.3 kb region between *hphMX* and the first essential gene (*PCM1*). Distributions were compared using the Fisher's exact test.

To determine the nature of the GCR events induced by CEB1-WT-1.7, we isolated a set of Can/5FOA-resistant colonies from independent cultures to avoid sibling events and analyzed their genomic DNA by Southern blot. The DNA was digested with a restriction enzyme cutting in the centromere proximal part of CEB1 and successively visualized with a CEB1 and a telomeric probe on the same blot. In the majority of colonies isolated in the WT strain background (29/31, 94%) it revealed a smeared CEB1 hybridizing band, which co-hybridized with the telomeric probe (Figure 3A). Similar events and proportion were found for the WT strain treated with Phen-DC_3_ (18/18), *pif1Δ* cells (18/19) (Figure 4C), and WT cells carrying the CEB1-Gmut-1.7 array (8/10 events). Thus, these GCR are likely telomere addition (telomere have variable length in the cell population) associated with a variable number of residual CEB1 motifs.

Analysis of the median length of the smeared band allowed us to roughly determining the number of remaining CEB1 motifs. In untreated WT cells, the events were evenly distributed along the 43 CEB1-WT motifs with the median telomere addition at the 25^th^ motif (Figure 3B). In contrast, upon treatment of WT cells with Phen-DC_3_, or deletion of *PIF1*, telomere addition sites shifted significantly toward small fragments, with a median of 11 (p-value = 6×10^−4^) and 15 (p-value = 9.1×10^−3^) motifs, respectively (Figure 3B). These results indicate that (i) irrespectively of the nature of the CEB1 array, most GCR events are telomere addition within CEB1, (ii) telomere addition can occur at numerous places within the CEB1 array thus leaving a variable number of CEB1 motifs, and (iii) impairing the ability of cells to unwind G-quadruplexes (Phen-DC_3_ and *pif1Δ*) is associated with an increased loss of CEB1 motifs.

To gain higher resolution mapping of the telomere healing events within the CEB1 motifs, we sequenced a set of CEB1-telomere junctions using Ion Torrent Next-Generation Sequencing technology after purification of appropriate DNA bands on agarose gel (see Materials and Methods). We identified the CEB1-Tel junctions from 15 untreated and 12 Phen-DC_3_-treated WT cells (Figure 3C and Figure S2, respectively).

Telomere additions occur mainly at regions of the CEB1 motif that exhibit limited homology to the yeast telomeric sequence. Precisely, 10/27 CEB1-telomere junctions lie in the longest sequence of homology between CEB1 and the telomeric sequence (GGGTGG) and 24/27 junctions have at least two nucleotides in common between CEB1 and the telomeric sequence (shown in blue in Figure 3C). This result is consistent with previous observations showing that for *de novo* telomere addition to occur, homology to telomeric sequence of 2-bp (TG, GG, and GT dinucleotide) is sufficient and that a longer homology facilitates telomere healing [30], [34], [35]. The fact that 62% of the telomere additions occur in, or at, the junction with the G-quadruplex-forming sequence of CEB1 (red lines Figure 3C) is consistent with the fact that 60% of the TG, GG, and GT dinucleotides overlap this sequence. The distribution of the telomere addition within the CEB1 motif is not significantly different in untreated and Phen-DC_3_-treated WT cells (Figure S2). Hence, although the Phen-DC_3_ treatment strongly increases the rate of GCR (Figure 2D) and affects the position of the telomere addition in the array (Figure 3B), the position of the CEB1-telomere junction remains unaffected and mainly lies in the G-quadruplex-forming sequence (Figure 3C and Figure S2).

Altogether, these results suggest that the G-quadruplexes present within the CEB1 array in conditions where the capacity of the cell to unwind G-quadruplexes is impaired (upon Phen-DC_3_ treatment or *PIF1* deletion) stimulate the formation of GCR associated with a decreased number of CEB1 motifs remaining in the final repair product.

### Rad51- and Rad52-dependent and -independent telomere additions in CEB1

Telomere healing may occur by *de novo* telomere addition to a 3′ ssDNA extremity, especially in the absence Pif1 [29], [34]–[36], leaving a specific pattern of telomeric sequences [34]. However, among the 27 junctions we sequenced, we do not notice any obvious addition of a particular pattern of telomeric sequence in CEB1. On the other hand, telomere addition could occur by capture of endogenous telomeric sequences by break-induced replication (BIR) [28], [37], [38]. We examined the effect of the deletion of the *RAD51* or *RAD52* genes that are required for BIR [37], [38] but not for direct telomere addition by telomerase. It causes a 2-fold decrease of the GCR formation in strains bearing CEB1-WT-1.7, with rates of 2.92×10^−7^ and 3.54×10^−7^ events/generation, respectively (Figure 3D). The extent of the decrease is similar (3- to 5-fold) upon Phen-DC_3_ treatment, with GCR rates of 1.13×10^−5^ and 7.12×10^−6^ events/generation in the *rad51Δ* and *rad52Δ* mutants, respectively (Figure 3D). Interestingly, the molecular analyses of the nature of the events provided additional information. We found that the drop of the GCR rate in the absence of Rad52 is associated with a specific decrease of GCRs by telomere addition within CEB1 (Figure S3) while the analysis of the CEB1-telomere junction sequences recovered from untreated or Phen-DC_3_-treated WT cells revealed the presence of SNPs around the junction in 4/6 strains (Figure 3E). These SNPs are found either in the telomeric sequence only, or both in the CEB1 and the telomeric sequence around the junction (Figure 3E). These intriguing observations suggest that in WT cells roughly half of the telomere healing events in CEB1 occur by BIR on an ectopic telomere sharing a region of limited homology with the CEB1 motif [28]. SNPs found at the junction may result from the correction of the heteroduplex formed between CEB1 and the telomeric sequence, and/or by misincorporation of nucleotides in the early BIR steps [39].

### The structure of the GCR, but not the rate, depends on the orientation of CEB1

CEB1 strands strongly differ with respect to their GC composition (GC-bias = 76.6%) and the density of TG/GG/GT dinucleotide (bias is 87%) that seeds GCR by telomere healing (Table 1). We examined the behavior of CEB1 placed in the opposite orientation (orientation C) relatively to the distal telomere (Figure 1B). Strikingly, in WT cells, the GCR rates of CEB1-WT-1.7 are similar in either orientation (6.97 and 7.47×10^−7^ events/generation)(Figure 4A) and alike the G-strand, the GCR rates increase according to the total size of the array (Figure S4). Similarly, although occurring at various absolute rates, there is no significant orientation-dependent difference in all the other strains and conditions that we assayed (Figure 4A and 4B, Table S3). Namely, in WT cells carrying the CEB1-WT-1.7 array treated with Phen-DC_3_ (3.65 and 1.66×10^−5^ events/generation), CEB1-WT-1.7 in *pif1Δ* cells (3.89 and 4.6×10^−4^ events/generation), CEB1-Gmut-1.7 in WT (2.77 and 2.07×10^−8^ events/generation) and *pif1Δ* cells (6.32 and 3.05×10^−6^ events/generation) nor in cells carrying HRAS1-0.7 in WT (8.48×10^−9^ and 1.1×10^−8^ events/generation,) and *pif1Δ* cells (3.68 and 3.2×10^−6^ events/generation) (Figure 4A and 4B, Table S3). Hence, both in the WT and *pif1Δ* cells, the GCR rates induced by CEB1-WT-1.7, CEB1-Gmut 1–7, and HRAS1-0.7 are not affected by the minisatellite orientation on the chromosome. However, the pattern of rearrangements in the G and C orientations is very different (Figure 3A and Figure 4C). In WT cells bearing CEB1-WT-1.7 in the orientation C, only 2/22 rearrangements are smears indicative of telomere healing. The DNA of two other colonies migrates at the size expected for an unaltered Chr. V. By PCR analysis of *CAN1* and *URA3*, we observed that clone 12 (Figure 4C) retained both genes. Sequencing identifies a mis-sense mutation in *URA3* (G411A) and a frameshift in *CAN1* (del595G). It might be a rare case of two independent mutagenic events but more likely a mutagenic fill-in synthesis by BIR [39], occurring in this case on the sister chromatid to restore a full-length chromosome V. The other clone has lost *CAN1* and *URA3*. Thus, it is a structural variant like the majority of events (19/22), which manifest themselves as discrete bands of various sizes. Among them, 15 hybridize with both the *hphMX* and the CEB1 probes (Figure 4C). The variable hybridization intensity of the CEB1 signal indicates that the amount of remaining CEB1 sequence in the rearranged chromosomes is different from one strain to another (for example, compare lanes 6 and 10 in Figure 4C). It is interesting to note that in some cases (4/18), two or more bands hybridizing both the CEB1 and *hphMX* probes are visible (clones 1–3, and 7). To gain more insights into the nature of these rearrangements, we analyzed clones 1–4 by pulse-field gel electrophoresis and Comparative Genomic Hybridization (CGH) (Figure S5). All exhibit an abnormal migration of Chr. V, while the rest of the karyotype appears normal (Figure S5A). As expected, CGH analysis revealed that the distal part of Chr. V containing *URA3* and *CAN1* is lost (Figure S5B). Furthermore, complex changes in copy number on other chromosomes are detected (details are reported in Figure S5). To be noticed, Ty1 elements are present in the vicinity of the breakpoints, suggesting that they are preferred sites for GCR [40]. Thus, contrary to the prominent telomere additions observed in the G orientation, GCR induced by CEB1 in the C orientation are diverse and complex, as observed among spontaneous GCR events [28], [40]. The similar rate but different product structures in the G and C orientations can be explained if they result from a similar initiating event but difference in repair; In the G orientation, BIR starting within CEB1 on a telomere substrate will process in the chromosomal distal direction and immediately heal the initiating lesion. In the C orientation, BIR on a telomere substrate will process in the proximal direction to copy the entire chromosome, thus leading to the formation of a dicentric molecule prone to secondary complex rearrangement(s) before stabilization [41].

Furthermore, to address the genetic requirements of these GCR events, we examined the role of the non-homologous end joining (NHEJ) and homologous recombination (HR) pathways. The GCR rate remains unchanged in the *dnl4Δ* mutant (Figure S6A) while we observed a small but significant 4-fold decrease of the GCR rate in the *rad51Δ* and *rad52Δ* mutants (Figure S6A). In the absence of Rad52, the remaining events are telomere additions (8/9 events) (Figure S6B) suggesting that the HR pathway plays a major role in the formation of the structural but not telomere addition events generated by CEB1 in the C orientation.

### The GCR rate induced by CEB25 depends on its ability to bind Cdc13

We next asked what could be the molecular reasons for the high GCR rate induced by CEB25 in orientation G, and the inability of Pif1 to suppress GCR induced by this construct in WT cells (Figure 1C and 1D). The GCR rate is not dependent on Rad52 (3.9×10^−4^ events/generation) and all events in WT cells (11/11) are telomere additions within CEB25 (Figure S7). Interestingly, we found that contrary to CEB1, the GCR rate induced by CEB25 strongly depends on its orientation: the inversion of CEB25 caused a 516-fold decrease of the GCR rate in WT cells (2.24×10^−7^ events/generation). In *pif1Δ* cells, the GCR rate of CEB25 in the orientation C was close to the “no insert” control strain (2.41×10^−6^ vs. 1.01×10^−6^ events/generation). This strong orientation-dependency prompted us to investigate the sequence composition of the CEB25 motif.

**Figure 5:**
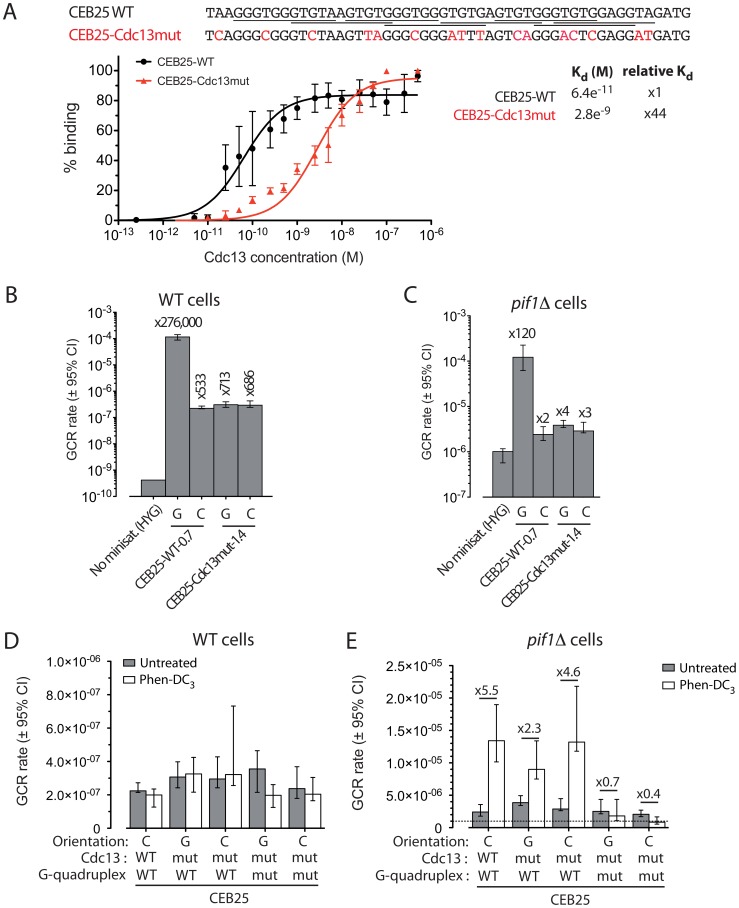
The high rate of GCR induced by CEB25 in an orientation-dependent manner relies on its high affinity Cdc13-binding sites. (A) Analyses of the Cdc13 affinity for CEB25 motifs. The sequences of the CEB25-WT and CEB25-Cdc13mut oligonucleotides used for the gel shift assay experiment are shown. The 11 nt high affinity Cdc13-binding sites (GTGTGGGTGTG, in which the 4 first nucleotides are critical) are underlined [42]. Mutations of the Cdc13-binding sites in CEB25-Cdc13mut are shown in red (16 mutations). The quantification of the proportion of the Cdc13-bound oligonucleotides of CEB25-WT (black) and CEB25-Cdc13mut (red) as a function of the Cdc13 concentration is shown. A second order binomial fit has been applied. Absolute K_d_ values ± SD and fold difference compared to the CEB25-WT motif are indicated. (B) GCR rates in the control “no minisatellite” WT strain (ORT6531), and WT cells bearing CEB25-WT-0.7 in the orientation G (ORT6558) and C (ORT6556), and CEB25-Cdc13mut-1.4 in the orientation G (ANT1181-1) and C (ANT1180-5). Other legends as in Figure 1C. (C) GCR rates in the control “no minisatellite” *pif1Δ* strain (ORT6568), and *pif1Δ* cells bearing CEB25-WT-0.7 in the orientation G (ORT6559-5) and C (ORT6557-1), and CEB25-Cdc13mut-1.4 in the orientation G (ANT1185-4) and C (ANT1184-1). Other legends as in Figure 1D. (D) GCR rates in WT cells bearing CEB25-WT-0.7 in the orientation C, CEB25-Cdc13mut-1.4 in the orientation G and C, or CEB25-Cdc13mut-Gmut-1.4 in the orientation G and C treated with 10 µM Phen-DC_3_ (white) or not (grey). (E) GCR rates in *pif1Δ* cells bearing CEB25-WT-0.7 in the orientation C (ORT6557-1), CEB25-Cdc13mut-1.4 in the orientation G (ANT1184-1) and C (ANT1185-4), or CEB25-Cdc13mut-Gmut-1.4 in the orientation G (ANT1187-1) and C (ANT1186-1) treated with 10 µM Phen-DC_3_ (white) or not (grey).

CEB25 has a GC content of 58% and exhibits an absolute GC-bias and GT/GG/TG dinucleotide bias (Table 1). Interestingly, it contains several consensus-binding sites for the 3′ telomeric overhang binding protein Cdc13 (GTGTGGGTGTG, in which the first 4 nucleotides are critical [42], underlined in Figure 5A) [43], [44]. Cdc13, together with Stn1 and Ten1, is a part of the CST complex involved in telomere capping and mutagenic DSB repair by addition of telomeric repeats at a 3′ ssDNA end [32], [45]–[47]. This unique feature, compared to CEB1 and HRAS1, led us to suspect that the recruitment of Cdc13 on CEB25 could be responsible for its GCR effect. To test this hypothesis, we conducted both *in vitro* and *in vivo* experiments. *In vitro*, we determined the affinity of the purified Cdc13 for the CEB25 motif upon gel shift assay (Figure 5A). Cdc13 binds with high affinity to the CEB25 motif (CEB25-WT), with a K_d_ = 6.4×10^−11^±10^−11^ M. Mutations of the Cdc13 binding sites present in the CEB25 motif (CEB25-Cdc13mut) resulted in a 44-fold lower affinity for Cdc13 (K_d_ = 2.8×10^−9^±3×10^−10^ M)(Figure 5A).

Then, to address the possibility that the high affinity of Cdc13 for CEB25 is responsible for the high GCR rate induced by this minisatellite only when the G-rich strand is in the same molecule than the distal telomere (and thus can be directly extended by telomerase), we constructed and introduced in yeast a 1.4 kb CEB25 allele mutated for its Cdc13-binding sites (CEB25-Cdc13mut-1.4, same motif as in Figure 5A) that kept the same GC content and did not change the G-triplets potentially involved in the G-quadruplex formation (see below). Remarkably, in the orientation G, this construct induced a GCR rate of 3.07×10^−7^ events/generation. This is 713-fold higher than in the “no minisatellite” control strain, and 377-fold lower than with CEB25-WT-0.7 in the same orientation (Figure 5B). Contrary to CEB25-WT, the GCR rate was not affected by the inversion of CEB25-Cdc13mut-1.4 (2.95×10^−7^ events/generation), indicating that the strong orientation dependency observed with CEB25-WT relies on the presence of the Cdc13-binding sites (Figure 5B). Additionally, in the absence of Pif1, CEB25-Cdc13mut-1.4 also shows a decreased GCR rate compared to CEB25-WT-0.7 in the orientation G (60-fold)(Figure 5C). Again, the GCR rate induced by CEB25-Cdc13mut-1.4 was similar in both the orientations G and C (3.86 and 2.89×10^−6^ events/generation, respectively), and close to the control *pif1Δ* strain (1.01×10^−6^ events/generation)(Figure 5C). Hence, the orientation-dependent and Pif1-independent behavior of CEB25-WT is associated with the ability of its motifs to bind the accessory telomerase subunit Cdc13 with high affinity.

### CEB25 does not induce G-quadruplex-dependent GCR

CEB25 contains a consensus G-quadruplex-forming motif (Table 1) that forms a monomorphic G-quadruplex whose structure has been recently solved by NMR [27]. To investigate the potential involvement of G-quadruplexes in the fragility of CEB25, we first examined the GCR rate of CEB25-Cdc13mut-1.4 in the WT and *pif1Δ* strains (mutations of the Cdc13 binding sites does not change the G-triplets involved in G-quadruplex formation). We found that GCR rates were (i) similar in these strains (Figure 5C), (ii) occurred at a low level comparable to CEB1-Gmut-1.7 (Figure 4B) and HRAS1 (Figure 1D) and (iii) lower than for CEB1-WT-1.7 (Figure 4B). To investigate the potential role of the CEB25 G-quadruplex forming sequences, we synthesized a CEB25 allele mutated for both the G-tracts and the Cdc13 binding sites (CEB25-Cdc13mut-Gmut-1.4). Clearly, the Phen-DC_3_ treatment of WT cells bearing CEB25-Cdc13mut-1.4 and CEB25-Cdc13mut-Gmut-1.4 alleles in both orientations yielded no increase of the GCR rates (Figure 5D). This did not depend on the absence of intact Cdc13 binding sites since the CEB25-WT-0.7 allele in the orientation C also remained insensitive to Phen-DC_3_ (Figure 5D). Rather, the G-quadruplex-forming and the G-mutated versions of CEB25-Cdc13mut exhibited exactly the same rates of GCR in WT cells. This absence of effect of Phen-DC_3_ contrasts with the 22- to 52-fold inductions observed with CEB1-WT upon WT cells treatment (Figure 4A). We then combined the deletion of *PIF1* to the Phen-DC_3_ treatment, conditions that yielded synergistic destabilization of CEB1 [18]. We observed a weak 5.5-, 2.3- and 4.6-fold induction of the GCR rates upon treatment of cells bearing CEB25-WT-0.7 in the orientation C, and CEB25-Cdc13mut-1.4 in the orientations G or C, respectively (Figure 5E). No induction was seen upon treatment of cells bearing the G-mutated version of CEB25-Cdc13mut (Figure 5E). These extreme conditions revealed a slight G-quadruplex-dependent GCR induction by CEB25.

### GC-rich and G-quadruplex-forming minisatellites cluster at chromosome ends in the human genome

**Figure 6:**
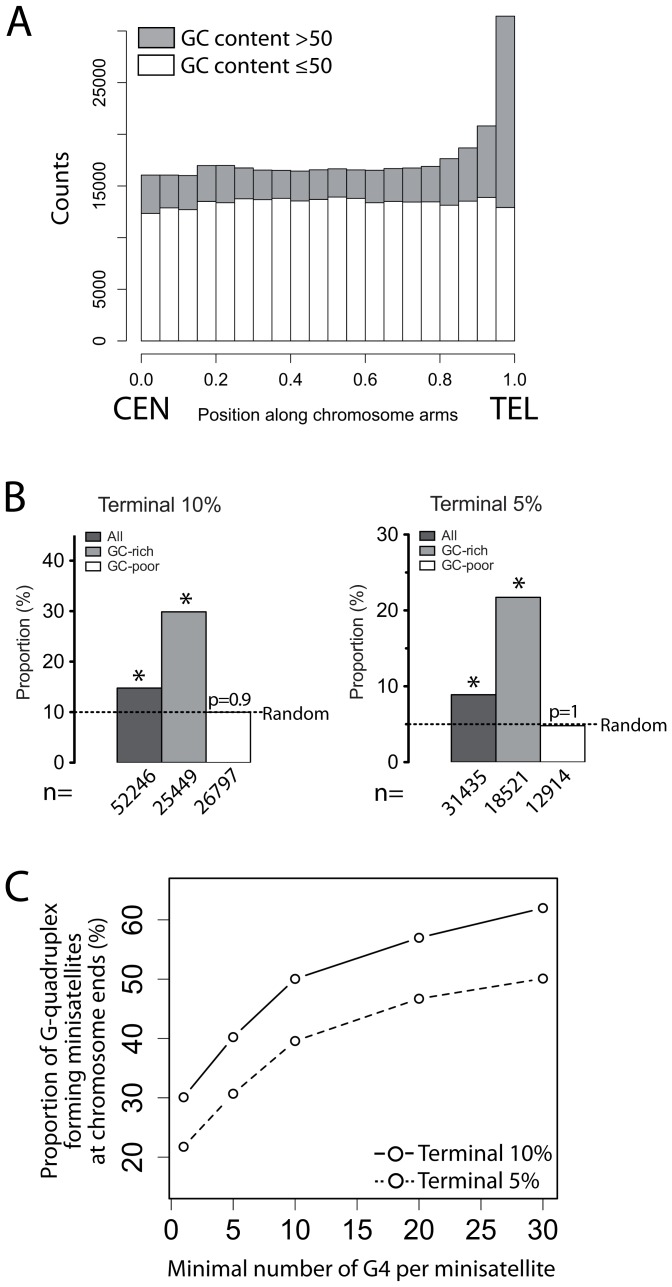
GC–rich and G-quadruplex-forming minisatellites are enriched at chromosome ends in the human genome. (A) Distribution of the human minisatellites along a normalized chromosome arm in the human genome. The centromere (CEN) is at 0, and the telomere (TEL) at 1. (B) Proportion of the GC-rich (GC content >50%), GC-poor (GC content ≤50%), and all the minisatellites in the terminal 10 and 5% on each chromosome arms. n indicates the number of minisatellites. A star (*) indicates a significant enrichment (all p-values <2e^−16^). No enrichment is observed for GC-poor minisatellites. (C) Proportion of minisatellites in the terminal 10% (continuous line) and 5% (dotted line) of chromosome arms as a function of the number of non-overlapping G-quadruplex-forming sequences per minisatellite.

Since the minisatellites studied here induced the formation of GCR, we wished to gain more insights into the GC-rich minisatellite representation and localization in the human genome. Using Tandem Repeat Finder [48], we determined a list of 353,460 minisatellites (Table S4). They are not evenly distributed along chromosome arms (Figure 6A) [20], being enriched in the 10 and 5% terminal arm regions (Figure 6B). Interestingly, it seems to relate to their GC-content since the 85,222 minisatellites (24%) that have a GC-content higher than 50% preferentially localize at the most terminal parts of the chromosome, whereas the other minisatellites with a lower GC-content are evenly distributed along the arms (Figure 6A and 6B). A similar bias has been previously reported for chromosome 22 [19]. Then, we examined the minisatellites having potential G-quadruplex-forming sequences. Five percent (18,906) of the minisatellites bear at least one G-quadruplex-forming sequence (see Materials and Methods), and 96% (18,191) of these G-quadruplex-forming minisatellites are GC-rich (Table S5). Among the 504 minisatellites that contain at least 30 G-quadruplex-forming sequences due to their tandem repeated structure, 60% (313/504) lie within the terminal 10% of chromosome arms, among which 80% (253/313) lie within the terminal 5%, while keeping a constant GC-content (Figure S8). Hence, GC-rich and G-quadruplex-forming minisatellites appear to preferentially cluster towards the chromosomal extremities (Figure 6C). The mutagenic behavior of HRAS1, CEB1 and CEB25 arrays in yeast described here raises the possibility that the human GC-rich minisatellites play a role in GCRs of the terminal part of human chromosomes.

## Discussion

In this study, we assayed the fragility of three GC-rich human minisatellites and mutant derivatives in *S. cerevisiae*. All these minisatellites stimulated the formation of GCRs but at rates varying by several orders of magnitude. We found that the rate depends on several intrinsic sequence features: the total number of repeats, the ability or not to form G-quadruplex secondary structures (case of CEB1) or to bind with high affinity the telomere ssDNA binding protein Cdc13 (case of CEB25). These features also explain their different levels of responsiveness to the Pif1 helicase controlling telomere elongation and G quadruplex unwinding. CEB1 and CEB25 are also differentially responsive to their orientation on the chromosome; it drastically affects the GCR rate of CEB25 but not HRAS1 or CEB1, and in all cases dictates the type of GCR (telomere addition versus other structural rearrangements). Thus, the behavior of these minisatellites is largely specific. We uncovered here their sequence features.

### Roles of Pif1 and Cdc13

Spontaneous GCR in WT cells occurs at a very low rate (10^−10^). It yields a variety of rearrangements that delete the non-essential distal chromosomal region and rescue the chromosome by telomere addition at breaks that contain limited homology to telomere-like seed sequences as well as through more complex genome rearrangements [28]. Two factors may increase the rate of GCR: an increased number of initiating lesions or defects in the repair pathways [28], [29]. Regarding the later possibility, as previously reported, we observed that Pif1 plays an important role in suppressing the formation of GCR by telomere healing [29], [30], [32], [49]. In all but one of our minisatellite insertions, GCR rates were increased by several orders of magnitude upon *PIF1* deletion. However, in sharp contrast, the extreme GCR rate stimulated by CEB25 in WT cells remained roughly the same in *pif1Δ* cells. This insensitivity to Pif1 depends on the orientation of CEB25 relative to the distal telomere (G-strand in the same orientation as the single-stranded telomere G-overhang is the most active) in agreement with the ability of the motif to bind the endogenous Cdc13 yeast protein with high affinity (Figure 5A and 5B). Clearly, the mutation of the three Cdc13-binding sites yields a ≈380-fold reduction in GCR, consequently abolishing the CEB25 orientation-dependent behavior. The simplest interpretation of these results is that the recruitment of Cdc13 to CEB25 is sufficient to overcome the suppressive effect exerted by Pif1 to prevent the recruitment of the telomerase [46]. This is consistent with the Pif1-independent *de novo* telomere addition at a long internal telomeric tract (TG)_81_ introduced near an unrepairable HO break [32]. In our assay, due to its motif sequence and its organization in tandem repeats, the human CEB25 minisatellite fortuitously resembles a pseudo-telomere.

### Role of G-quadruplexes

On the other hand, the HRAS1, CEB1, CEB1-Gmut, CEB25-Cdc13mut and CEB25-Cdc13mut-Gmut tandem arrays devoid of Cdc13 binding sites also induce GCR but at various rates and in an orientation-independent manner. Among the parameters potentially involved in the fragility of CEB1, its ability to form G-quadruplexes appeared as an important destabilizing feature. Compared to the CEB1-Gmut-1.7 construct, the G-quadruplex-prone CEB1-WT-1.7 allele stimulates the GCR rate in WT cells 30-fold (Figure 2B) and accordingly the conditions that shift the equilibrium toward the folded G-quadruplex state increase the GCR rate : 52-fold upon treatment with the G-quadruplex stabilizing ligand Phen-DC_3_ and 558-fold in the absence of the G-quadruplex unwinding helicase Pif1 (Figure 2B and 2C, Figure 3D and 3E). However, it should be emphasized that a predictive G-quadruplex-dependent phenotype cannot be safely ascertained from the presence of a consensus G-quadruplex motif in a given sequence, nor its ability to form stable G-quadruplexes *in vitro*. Indeed, contrary to CEB1, the CEB25 array did not responded *in vivo* to the three conditions that affect G-quadruplex-dependent events (G quadruplex motif mutation, treatment with PhenDC_3_ or Pif1 deletion) except slightly, when combining the Phen-DC_3_ treatment to the *PIF1* deletion (Figure 5E). This synergistic combination previously observed for CEB1 [18] appears as an extreme hypersensitive condition that may lead to the rare accumulation of unprocessed CEB25 G-quadruplexes. The distinct behavior of CEB1 and CEB25 may rely on different conformations of their respective G-quadruplexes affecting their folding and/or their processing *in vivo*.

### Role of the repeats number

Besides sequence affinity to Cdc13 and potential to form G-quadruplexes, a third aggravating factor stimulating the GCR rate is the total number of motifs. Thanks to the sensitivity of this GCR assay, we found that the GCR rate of CEB1 arrays increased exponentially with the number of motifs without an apparent threshold in both WT and *pif1Δ* cells (Figure 2F and 2G). Interestingly, a similar exponential relationship between the number of motifs and the propensity of the triplex-forming (GAA)_n_ repeats [50] to form GCRs [51] and expansions [52] has also been reported in yeast. It suggests the intriguing possibility that the capacity of tandem arrays to form secondary structures is a relevant feature. Along this line, we know that a tandem of two and three CEB25 motifs is able to form a pearl-necklace monomorphic G-quadruplexes structure [27]. If CEB1 is also able to form a pearl-necklace G-quadruplexes structure, the size-dependent exponential increase of the GCR rate may reflect the cooperative behavior between the CEB1 motifs to fold into G-quadruplexes. Mechanistically, we recently reported that the CEB1 G-quadruplex prone array perturbs replication and lead to expansion and contraction events [17]. As we proposed, the blockage of the DNA polymerase(s) at the first G-quadruplex may be sufficient to trigger the accumulation of ssDNA between the replication forks and the polymerase and thus enhance the formation of G-quadruplexes per cell and per molecule in a manner related to the total number of repeats. This situation may be similar to the Pol2 slowdown observed at single G-quadruplex-forming motifs under treatment of Pif1-deficient cells with the replication inhibitor hydroxyurea [53].

### Other factors

In addition to the effect of G-quadruplexes, other non B-DNA secondary structures can be the source of sequence fragility [8]. However, we found that the HRAS1-0.7 and CEB25-Cdc13mut-Gmut-1.4 minisatellites, devoid of potential G-quadruplex or other secondary structures, also stimulated the GCR rate by 20- and 700-fold in WT cells, respectively. In addition, once the G-quadruplex-forming capacity of CEB1 was removed by site-directed mutations, we noted that the CEB1-Gmut-1.7 construct was still able to stimulate GCRs at a substantial level (≈2×10^−8^ events/generation), approximately 60-fold higher than in the control WT strain. Similarly, the structure-free (ATTCT)_n_ microsatellite has been reported recently to induce chromosomal fragility in WT yeast cells, which increase with the number of motifs [54]. However, the slope of this length-dependent effect could not be derived from these experiments since only two different allele sizes have been assessed [54]. The analysis of CEB1-Gmut allele of various lengths (23–70 motifs) revealed a length-dependent fragility in WT cells in an almost linear manner (multiplying the number of motifs by two increased the GCR rate by 4), in sharp contrast with the exponential slope observed with CEB1-WT (Figure 2F). This difference suggests that the G-quadruplex-independent fraction of the CEB1 fragility does not involve a cooperative behavior between the motifs. What remaining sequence properties could account for this structure-independent fragility? The GC-richness *per se* can be invoked, since it has been shown to slowdown DNA polymerases *in vitro* [55]. In the case of our minisatellites, however, three reasons argue against its essential role. First, with similar size arrays, the GCR induction is not clearly correlated to the GC-richness: HRAS1 (GC = 67%) and CEB1-Gmut (72%) both stimulated the GCR rate ≈20-fold compared to the no insert strain, but ≈35-fold less than CEB25-Cdc13mut (GC = 56%). Second, the *hphMX* insert, whose size and GC content is similar to CEB25-Cdc13mut-1.4 (≈58%), did not stimulate GCR above the no-insert control strain. And third, the density of TG/GG/GT dinucleotides that can seed telomere addition is similar in the CEB25-Cdc13mut, HRAS and hphMX insertions (Table 1). These observations suggest that the GC-richness is not *per se* the determinant triggering GCR. The remaining shared feature of these sequences is their organization in tandem. By itself, it may perturb the normal progression of replication due to the high local concentration of homologous templates or create long range specific chromatin structures that might be processed at the expense of maintaining genome stability.

### Comparison between minisatellite-induced GCR and internal rearrangement frequencies

In addition to inducing truncated arrays and motifs by GCR, CEB1 also varies in size by increasing or decreasing the total number of motifs via SDSA and/or template switch without involving the flanking regions [16]–[18]. These events are extremely frequent, being detected in 8.3 and 20.3% of the cells upon deletion of *PIF1* or Phen-DC_3_ treatment, respectively (Table 2). This is 100–1000 fold higher than the GCR rates (3×10^−4^ and 3.6×10^−5^ events/generation, respectively) of the same construct. Thus quantitatively, expansion/contraction is the major outcome of CEB1 instability with the advantage to avoid the formation of potentially detrimental structural rearrangements. This is in agreement with numerous reports that compared internal rearrangements and GCR induced by different microsatellites [2], [56]–[58]. Mechanistically, since the presence of CEB1 perturbs replication [17], GCR events might result from the rare situations in which the template directed intra-motif interactions failed, allowing break-induced replication on an ectopic telomere sequence [51] or the recruitment of the telomerase to act. Consistent with a role of the homologous recombination pathway, we observed that the deletion of the *RAD51* or *RAD52* genes yield a ≈4-fold decrease of the GCR rate (Figure 3D and Figure S4A). This is true in both orientations although the nature of the GCR events is different. The insufficient absolute frequency of GCR events (<10^−4^) prevented us to determine whether or not the variation of the GCR rates were compensated by an increase of the expansion/contraction events that can be detected by Southern blot analyses of individual or small pool of colonies.

### Role and consequences of the preferential location of the human GC–rich minisatellites in chromosomal subtelomeric regions

Chromosomal rearrangements are potentially detrimental for cell functions and are the source of genetic diseases and cancer. Remarkably, subtelomeric regions are highly dynamic in primate and altered in approximately a third of the human pathologies involving chromosomal rearrangements [21], [23], [24], [59], [60]. However, the factors involved in the high propensity of these regions to break and rearrange have not been identified. The intergenic CEB1 and HRAS1, as well as the intronic CEB25 minisatellites assayed here are located 400 kb–1.4 Mb away from the telomeres (Table 1), representative of the enrichment for GC-rich and the G-quadruplex-forming minisatellites at chromosome terminal regions in the human genome (Figure 6A and 6B). In yeast, the orientation does not affect the fragility *per se* but the nature of the GCR. Hence, given the high number of GC-rich minisatellites clustering at chromosome ends in the human genome irrespectively of their orientation, these sequences are likely implicated in the generation of the various subtelomeric rearrangements [61], [62]. But why these harmful sequences are massively present in the human genome? And what could be the reasons of their terminal clustering? A positively selected function could be to signal defects in replicating G-quadruplex-forming sequences [17], [53]. In this regard, the arrangement in tandem of G-quadruplex-forming motifs presents at least two advantages. First, they would act as severe “tandem of problems” for replication machinery as revealed by their exponential size-dependent fragility. Hence, cells with a decreased ability to remove G-quadruplexes will experience replication difficulties preferentially at these G-quadruplex-forming minisatellites rather than at unique sequences present throughout the genome and enriched in proto-oncogenes [17], [53], [63]. Second, owing to the higher local concentration of homologous template compare to unique sequences, they will preferentially undergo internal rearrangements rather than inducing structural variations. Thus, we envision that GC-rich and G-quadruplex-forming minisatellites help signaling deficient replication machineries, and their clustering at chromosome ends and repetitive nature overall limit the potential formation of detrimental structural rearrangements.

## Materials and Methods

### Strains

The genotypes of the *Saccharomyces cerevisiae* strains (S288C background) used in this study are reported in Table S1. All strains have been derived from RDKY3615 (WT strains) [28] or RDKY4399 (*pif1Δ* strains) [29] by regular lithium-acetate transformation. Correct insertion of the hphMX cassette with or without minisatellite at *NPR2* (position 804, BamHI site), as well as the minisatellite size, have been verified by Southern blot. The CEB1-WT-1.7 and CEB1-Gmut-1.7 minisatellites have been synthesized previously [16]. Contractions and expansions of these minisatellites have been generated during the insertion procedure at the *NPR2* locus and are thus independent clones. The CEB25-WT-0.7, CEB25-Cdc13mut-1.4, and CEB25-Cdc13mut-Gmut-1.4 minisatellites have been synthesized *in vitro* using PCR-based method as previously described [16]. The HRAS1 minisatellite of 0.7 kb (HRAS1-0.7) has been obtained from P37Y8 (gift from D. Kirkpatrick) [64]. The motifs of the minisatellites used in this study are presented in Table S2. Deletion of *RAD51*, *RAD52*, and *DNL4* has been performed by transformation of the corresponding KMX cassettes amplified from the EUROSCARF deletants collection [65]. Primer sequences are listed in Table S6.

### Media

Liquid synthetic complete (SC) and solid Yeast-Peptone-Dextrose (YPD) media have been prepared according to standard protocols [66]. Plates containing Canavanine (Sigma-Aldrich) and 5FOA (Euromedex) have been prepared according to standard protocols [67] with minor differences: because *npr2Δ* cells exhibit a decreased resistance to acidic pH (<4.0) [68] we adjusted the pH to 4.5–4.8 (instead of 2.8–3.0) and compensated the decreased penetration of 5FOA at this pH by using it at a slightly higher concentration (≈1.5X instead of 1X). SC liquid media containing Phen-DC_3_ (1, 5, or 10 µM) and Phen-DC_6_ (1 or 5 µM) have been prepared as previously described [18].

### Fluctuation analysis

The GCR rate has been determined by fluctuation analysis of 5FOA and canavanine-resistant cells. A ura+ colony is used to inoculate at least 10 independent cultures at a concentration of ≈10^2–3^ cells/mL in 2–50 mL of SC media and grown with shacking at 30°C. When they have reached saturation (2 days), cells are spread on 5FOA/canavanine-containing plates and on YPD plates. A maximum of 10^8^ cells was spread on 85 mm plates, and 10^9^ cells on 145 mm plates. The number of cells spread was adjusted in order not to exceed 100 colonies per plate. For G-quadruplex ligands-containing SC media, cells undergo an overnight preculture in SC prior to inoculation with the ligand, and are grown at 30°C up to saturation. For *pif1Δ* cells bearing CEB1-WT, which exhibit an inherently high level of CEB1 internal rearrangements, which can influence the GCR rate (strains ORT6543-1, ORT7153-9, and ORT6592-22), the size of the parental minisatellite is determined by Southern blot from individual colonies plated on YPD. The colonies bearing the parental size of CEB1-WT are directly spread on YPD and 5FOA/Can-containing plates without additional liquid culture. After 4 days at 30°C, the number of 5FOA/Can-resistant colonies (r) is counted, as well as the total number of viable cells spread (N_t_) derived from the number of colonies formed on YPD. The GCR rate (M) as well as the upper and lower 95% confidence intervals (95% CI) have been calculated from r and N_t_ with Falcor [69] using the Lea and Coulson method of the median. For each strain and condition, 10 to 45 independent cultures have been performed, in at least two independent experiments. The rates, 95% confidence intervals, and the number of independent cultures performed are listed in the Table S3.

### CEB1 instability measurement

Colonies grown on YPD plates after the 2 days culture in SC media are inoculated in 96-well megaplaque for 24–48 hours. Pools of 4–16 colonies were made right before DNA extraction. DNA was digested with XbaI/EcoNI (leaving 414 bp of flanking sequence) and migrated O/N in a 0.8% agarose-TBE 1X gel at 50 V. Digestion products were analyzed by Southern blot using a CEB1 radiolabeled probe. Blots were scanned using a Storm Phosphorimager (Molecular Dynamics) or a Typhoon Phosphorimager (GE Healthcare), and quantified using ImageQuant 5.2 as described in [18].

### Analysis of the nature of the GCR

In order to avoid sibling events, DNA of 5FOA/Canavanine-resistant colonies from separate cultures is extracted, digested using either SacI or XbaI, and migrated in a 0.8% agarose-TBE 1X gel overnight at 50 V. Digestion products were analyzed by Southern blot as described previously using a radio-labeled CEB1, hphMX (from pAG34), or telomeric (from pCT300) probe. The position of the telomere addition is estimated by measuring the size of the center of the smear, and subtracting both 50 bp of flanking sequence plus the mean telomere size (300 bp in WT cells and 400 bp in *pif1Δ* cells [30]).

### Sequencing of CEB1-telomere junctions

DNA of colonies bearing a CEB1-telomere smear identified by Southern blot was digested by XbaI and migrated in a 0.8% agarose-TBE 1X gel overnight at 50 V. After staining of the DNA with BET, the DNA fragments containing the CEB1-telomere junction were cut and extracted from the gel using the Nucleospin Extract II (Macherey-Nagel) kit. Fragments were quantified, pooled, and precipitated. Samples were prepared for Ion Torrent Personal Genome Machine (PGM, Applied Biosystems). Sequencing has been performed according to manufacturer instructions on a 314R chip. Reads have been validated and aligned on the S288c genome (R64-1-1, 2011-02-03) and custom CEB1-telomere templates using the in-built Torrent Suite 1.5.1. Reads matching both the CEB1 and the telomeric sequences have been isolated and analyzed manually using Tablet 1.11.11.01 [70] and Microsoft Excel 2007.

### *In silico* minisatellite analysis

The list of minisatellites (motif comprised between 10 and 100 bp) and their associated characteristics has been obtained form the Tandem Repeat Database [71] (list generated on the 2010-10-31 by the TRF algorithm [48] on the Homo Sapiens hg19 release). Overlapping duplicates of the same repeat due to uncertainties in the algorithm have been eliminated. The human minisatellites are listed in Table S4. The number of non-overlapping G-quadruplex-forming sequences per minisatellite have been determined using R software. The custom algorithm searches for 4 runs of 3 Gs in a window of 30 nt, with a minimal loop size of 1 nt, and consequently a maximal loop size of 16 nt [72]. They are listed in the Table S5.

### Cdc13 gel shift assay

A full length version of CDC13 WT was cloned into a pYES2 vector and expressed as a fusion with a C-terminal tag consisting of a 8 glycine linker, 5 strepII-tags (IBA, Germany) and a HAT-tag (Clontech). Cdc13 overexpression was induced in 2% galactose for 16 hours at 30°C according to the method described by P.M. Burgers [73]. Briefly, after grinding cell pellets in liquid nitrogen, the lysate was clarified from DNA by precipitation in 0.1% polyethyleneimine, and the proteins were precipitated with ammonium sulfate at 60% saturation. After resuspension in 50 mM Tris pH 8.0, 300 mM NaCl, 10% glycerol, the soluble fraction was loaded successively on a streptactin column (IBA, germany) followed by a Talon column (Clontech). Purified protein was dialysed against storage buffer 2X without glycerol, and concentrated and stored at −80°C in 1x storage buffer (25 mM tris-HCl pH 8.0, 250 mM NaCl, 0.5 mM DTT, 50% Glycerol). This procedure yielded homogeneous CDC13 estimated more than 90% pure by coomassie blue staining after protein separation by SDS-PAGE.

Gel shift was carried out by incubating 20 pM of the 52-mer CEB25 WT oligonucleotide or the 52_mer-Cdc13mut version, end-labeled at the 5′ end using γ-ATP and T4 polynucleotide kinase, with indicated amount of CDC13, in the following buffer: 5 mM Tris pH 8.0, 2.5 mM MgCl_2_, 0.1 mM EDTA, 2 mM DTT, 0.1 µg/µl BSA (NEB), 50 mM NaCl, 0.2 M LiCl. After incubation at room temperature for 30 minutes, binding reactions were supplemented with 3% Ficoll and run on a 6% native polyacrylamide gel (37.5∶1 acrylamide/polyacrylamide ratio), at 4°C and 8 V/cm. Gels were dried on DE81 paper and quantified using a Typhoon phosphorimager. Data were fitted to a one-site-specific binding equation (Y = Bmax*X/(Kd+X)) using Prism software (Graphpad), yielding R^2^ values for goodness of fit of 0.91 and 0.95 for CEB25-WT and CEB25-Cdc13mut, respectively.

### Statistical analysis

Statistical tests have been performed with R software 2.13.1 [74] or Graphpad Prism 5.0b. The α-cutoff for statistical significance was set to 0.05. Rearrangement frequencies of CEB1 have been compared using a two-tailed Fisher's exact test. Correlation between the number of CEB1 motifs and the rate of GCR has been determined using the Spearman correlation test. GCR rates, as well as the distributions of the position of telomere addition in the CEB1 array have been compared using a non-parametric test (Mann-Whitney-Wilcoxon, two-tailed). A one-tailed χ^2^ test has been used to determined the enrichment of minisatellites in the 10 and 5 terminal percent of chromosome arms.

## Supporting Information

Figure S1 The increased GCR rate in WT cells bearing CEB1-WT upon treatment with Phen-DC_3_ can not be explained by a better growth rate of cells having performed a GCR in presence of Phen-DC_3_ 10 µM. Growth of WT cells bearing CEB1-WT-1.7 in the orientation G (WT, ORT6542-6) and a derived strain bearing a CEB1 minisatellite fused to a telomere (GCR) have been monitored by measurement of the optical density at 600 nm (OD600) as described in [18]. The generation time has been measured during the exponential growth phase, between the 4 and 8 h time points. Error bars show SD (n = 3).
(EPS)

Figure S2 Analysis of the telomere addition site in the CEB1-WT motif in Phen-DC_3_-treated WT cells (ORT6542-6). Each CEB1-telomere junctions have been obtained from 12 5FOA/Can-resistant colonies from independent cultures. Other legends as in Figure 3C.
(EPS)

Figure S3 A subset of the telomere additions in CEB1 depends on Rad52. Proportions of the different types of GCR characterized by Southern blot analysis in WT (ORT6542-6) and *rad52Δ* (ORT7310-2) cells bearing CEB1-WT-1.7 in the orientation G. Other legends as in Figure 4D.
(EPS)

Figure S4 Size effect of CEB1-WT in wild-type and *pif1Δ* cells, in the orientations G and C.
(EPS)

Figure S5 Analysis of the nature of GCR obtained in WT cells bearing CEB1-WT-1.7 in the orientation C by pulse-field gel electrophoresis and CGH. The 5FOA/Can-resistant clones analyzed correspond to clones 1–4 in Figure 4C. (A) Analysis by pulse-field gel electrophoresis of the DNA of the parental 5FOA/Can-sensitive strain (WT, ORT6591-1) and of 4 5FOA/Can-resistant clones. The left panel shows the karyotype (BET staining). The right panel is a Southern blot revealing the Chr. V with a probe directed against the *FCY2* gene (see Text S1). The 4 clones have an abnormal migration pattern for the Chr. V. Clones 1 and 4 exhibit a single major band corresponding to the Chr. V at ≈540 kb and ≈760 kb, respectively. Clones 2 and 3 exhibit multiple faint bands. (B) Analysis by CGH of clones 1–4. The log_2_ of the ratio of the 5FOA/Can-resistant clone signal to the WT strain signal is plotted (see Text S1). Chromosomes presenting detectable loss (red, log_2_<0) or gain (green, log_2_>0) of copy number are shown for each clone. The mean increase in copy number, as well as the starting position of the last affected probe, is indicated. In all clones the terminal region of the left arm of the Chr. V, up to the position 34,915 (5′ of *NPR2*), is lost. No other copy number variation is detected in clone 1. Thus, the rearrangement is a truncation of the Chr. V (size ≈540 kb) by telomere addition in CEB1, consistent with the size measured by pulse-field gel electrophoresis and the smear observed by Southern blot (Figure 4C). The origin of the upper band hybridizing CEB1 and *hphMX* remains unexplained. In addition to the deletion of the terminal 35 kb of the Chr. V, clones 2–4 exhibit gains of terminal chromosome regions. Clone 2 have an increased copy number of 162 kb of the ch.III and 80 kb of the Chr. V. Clone 3 has an increased copy number of 93 kb of the ch.XVI, 80 kb of the left arm of the Chr. V and 120 kb of the right arm of the Chr. V. Clone 4 has an increased copy number of 80 kb and 120 kb of the left and the right arm of the Chr. V, respectively. The detected increase in copy number ranged from 1.15 to 1.9. FACS analysis shows that all the clones remained haploid. Thus, these colonies are mosaic for the various rearrangements of the Chr. V observed by Southern blot and pulse-field gel electrophoresis, suggesting that 5FOA/Can-resistant cells kept rearranging their altered Chr. V after plating on 5FOA/Can-containing plates. These duplicated regions are all terminal and the internal breakpoints fall in close proximity to Ty1 elements, indicated by red arrows. Our interpretation is that the lesion initiated in CEB1 is repaired by BIR on a telomere and creates a dicentric chromosome. It subsequently yields internal breakages and ty1-mediated rearrangements [41].
(EPS)

Figure S6 GCR are mainly Rad51- and Rad52-dependent translocations in WT cells bearing CEB1-WT-1.7 in the orientation C. (A) GCR rates in WT (ORT6591-1), *dnl4Δ* (ORT7309-3), *rad51Δ* (ORT7191-3), and *rad52Δ* (ORT7312-5) cells bearing CEB1-WT-1.7 in the orientation C upon treatment (right) or not (left) with 10 µM of Phen-DC_3_. Other legends as in Figure 1C. (B) Proportions of the different types of GCR characterized by Southern blot analysis in WT (ORT6591-1) and *rad52Δ* (ORT7312-3) cells bearing CEB1-WT-1.7 in the orientation C. Other legends as in Figure 4D.
(EPS)

Figure S7 Nature of the GCR induced by CEB25-WT-0.7 in the orientation G in WT cells. The top panel schematically represents the genomic region surrounding CEB25 with the *Sac*I restriction site and the *hphMX* (blue) probe used to study the rearrangements of the region. The sizes expected for the unaltered region or in the case of a telomere addition within CEB25 are shown. In the latter case, the band is expected to produce a smear of 2.1 kb (flanking sequence of 1.8 kb and ≈300 bp of telomere) plus the size of the remaining CEB25 sequence. The bottom panels show the rearrangements present in 11 5FOA/Can-resistant clones obtained from independent cultures of WT cells bearing CEB25-WT-0.7 in the orientation G, and in the parental 5FOA/Can-sensitive strain (WT, ORT6558). All rearrangements migrate as smears. The size is comprised between 2.2 and 2.6 kb (clone 11 and 9, respectively), consistent with a telomere addition event within CEB25 leaving between 0.1 and 0.5 kb of CEB25 sequence, respectively. M_1_ and M_2_ = Size markers.
(EPS)

Figure S8 The number of G-quadruplex forming sequence per minisatellite does not affect The GC-content of G-quadruplex-forming minisatellites. Error bars show SD.
(EPS)

Table S1 Strains used in this study.
(PDF)

Table S2 Minisatellites used in this study.
(PDF)

Table S3 GCR rates measured in the untreated cells (untreated sheet) and in cells treated with G-quadruplex ligands (Phen-DC sheet). The upper and lower 95% confidence intervals, as well as the number of independent cultures performed (n) are indicated. The number after the minisatellite name (CEB1-WT-) indicates its size in kb. The number of motifs has been determined by sequencing of the array for CEB1-Gmut-1.7 [16], HRAS1-0.7, CEB25-WT-0.7, CEB25-Cdc13mut-1.4 and CEB25-Cdc13mut-Gmut-1.4, and estimated based on their size for the various CEB1-WT and CEB1-Gmut alleles.
(PDF)

Table S4 Human minisatellites (motif comprised between 10 and 100 nt) determined using Tandem Repeat Finder (see Material and Methods) [48], [71]. Information concerning the position, motif size, copy number of the motif in the reference hg19 release, GC bias between the strands, total array length, and GC content of each minisatellite is provided.
(TXT)

Table S5 G-quadruplex forming human minisatellites. In addition to the basic information provided in Table S4, this table give the number of non-overlapping potential G-quadruplex-forming sequences for each minisatellite when searching for 4 runs of 3 Gs in a maximum of 30, 40, 50, or 100 bp sliding window (G4< = 30, G4< = 40, G4< = 50, G4< = 100, respectively)(see Materials and Methods). The number of G-quadruplexe-forming sequence per motif is also provided for the search performed with each window (last 4 lanes). The column “G4strand” indicates if the G-quadruplex-forming sequence was present in the Watson strand (G) or the Crick strand (C). Analyses presented in the Results section and in Figure 6 have been performed using data obtained with the most stringent search parameter (window ≤30 bp).
(TXT)

Table S6 Primers used in this study. Sequences are oriented 5′ to 3′.
(PDF)

Text S1 Supplementary Methods.
(DOCX)
