# Supplementary material for: Stimulation of Gross Chromosomal Rearrangements by the Human CEB1 and CEB25 Minisatellites in Saccharomyces cerevisiae Depends on G-Quadruplexes or Cdc13
Source: PLoS Genet. 2012 Nov 1;8(11):e1003033. doi: 10.1371/journal.pgen.1003033 (PMC3486850; doi:10.1371/journal.pgen.1003033)
Supplement: Table S1 — Strains used in this study. (PDF) [file pgen.1003033.s009.pdf]

Table S1

Strain	Relevant genotype	Source
RDKY3615	MAT a, *ura3-52, his3Δ200, leu2Δ1, trp1Δ63, lys2ΔBgI, hom3-10, ade2Δ1, ade8, hxt13::URA3*	ref. 28
RDKY4399	*pif1::KMX*	ref. 29
ORT6531	*npr2::hphMX*	This study
ORT6568	*pif1::KMX, npr2::hphMX*	This study
ORT6542-8	*npr2::hphMX-CEB1-WT-0.8 orientation G*	This study
ORT6542-5	*npr2::hphMX-CEB1-WT-1.4 orientation G*	This study
ORT6542-6	*npr2::hphMX-CEB1-WT-1.7 orientation G*	This study
ORT6542-4	*npr2::hphMX-CEB1-WT-1.9 orientation G*	This study
ORT6542-1	*npr2::hphMX-CEB1-WT-2.7 orientation G*	This study
ORT6591-9	*npr2::hphMX-CEB1-WT-0.2 orientation C*	This study
ORT6591-4	*npr2::hphMX-CEB1-WT-0.45 orientation C*	This study
ORT6591-3	*npr2::hphMX-CEB1-WT-0.8 orientation C*	This study
ORT6591-8	*npr2::hphMX-CEB1-WT-1.1 orientation C*	This study
ORT6591-1	*npr2::hphMX-CEB1-WT-1.7 orientation C*	This study
ORT6543-10	*pif1::KMX, npr2::hphMX-CEB1-WT-0.16 orientation G*	This study
ORT7154-2	*pif1::KMX, npr2::hphMX-CEB1-WT-0.28 orientation G*	This study
ORT7154-7	*pif1::KMX, npr2::hphMX-CEB1-WT-0.76 orientation G*	This study
ORT7154-3	*pif1::KMX, npr2::hphMX-CEB1-WT-0.88 orientation G*	This study
ORT6543-4	*pif1::KMX, npr2::hphMX-CEB1-WT-1.4 orientation G*	This study
ORT6543-1	*pif1::KMX, npr2::hphMX-CEB1-WT-1.7 orientation G*	This study
ORT6541-1	*pif1::KMX, npr2::hphMX-CEB1-WT-0.16 orientation C*	This study
ORT6592-1	*pif1::KMX, npr2::hphMX-CEB1-WT-0.4 orientation C*	This study
ORT6592-4	*pif1::KMX, npr2::hphMX-CEB1-WT-0.96 orientation C*	This study
ORT6592-18	*pif1::KMX, npr2::hphMX-CEB1-WT-1.2 orientation C*	This study
ORT6592-22	*pif1::KMX, npr2::hphMX-CEB1-WT-1.4 orientation C*	This study
ORT7153-9	*pif1::KMX, npr2::hphMX-CEB1-WT-1.7 orientation C*	This study
ORT7189-1	*rad51::KMX, npr2::hphMX-CEB1-WT-1.7 orientation G*	This study
ORT7191-3	*rad51::KMX, npr2::hphMX-CEB1-WT-1.7 orientation C*	This study
ORT7309-3	*dnl4::KMX, npr2::hphMX-CEB1-WT-1.7 orientation C*	This study
ORT7310-2	*rad52::KMX, npr2::hphMX-CEB1-WT-1.7 orientation G*	This study
ORT7312-5	*rad52::KMX, npr2::hphMX-CEB1-WT-1.7 orientation C*	This study
ORT6550-2	*npr2::hphMX-CEB1-Gmut-1.7 orientation G*	This study
ORT6548	*npr2::hphMX-CEB1-Gmut-1.7 orientation C*	This study
ORT6551-1	*pif1::KMX, npr2::hphMX-CEB1-Gmut-1.7 orientation G*	This study
ORT6549	*pif1::KMX, npr2::hphMX-CEB1-Gmut-1.7 orientation C*	This study
ORT7182	*npr2::hphMX-HRAS1-0.7 orientation G*	This study
ORT7183	*npr2::hphMX-HRAS1-0.7 orientation C*	This study
ORT7322	*pif1::HIS3, npr2::hphMX-HRAS1-0.7 orientation G*	This study
ORT7323	*pif1::HIS3, npr2::hphMX-HRAS1-0.7 orientation C*	This study
ORT6558	*npr2::hphMX-CEB25-WT-0.7 orientation G*	This study
ORT6556	*npr2::hphMX-CEB25-WT-0.7 orientation C*	This study
ORT7344	*rad52::KMX, npr2::hphMX-CEB25-WT-0.7 orientation G*	This study
ANT1181-1	*npr2::hphMX-CEB25-Cdc13mutG4wt-1.4 orientation G*	This study
ANT1180-5	*npr2::hphMX-CEB25-Cdc13mutG4wt-1.4 orientation C*	This study
ANT1183-1	*npr2::hphMX-CEB25-Cdc13mutG4mut-1.4 orientation G*	This study
ANT1182-1	*npr2::hphMX-CEB25-Cdc13mutG4mut-1.4 orientation C*	This study
ORT6559-5	*pif1::KMX, npr2::hphMX-CEB25-WT-0,7 orientation G*	This study
ORT6557-1	*pif1::KMX, npr2::hphMX-CEB25-WT-0,7 orientation C*	This study
ANT1185-4	*pif1::KMX, npr2::hphMX-CEB25-Cdc13mutG4wt-1.4 orientation G*	This study
ANT1184-1	*pif1::KMX, npr2::hphMX-CEB25-Cdc13mutG4wt-1.4 orientation C*	This study
ANT1187-1	*pif1::KMX, npr2::hphMX-CEB25-Cdc13mutG4mut-1.4 orientation G*	This study
ANT1186-1	*pif1::KMX, npr2::hphMX-CEB25-Cdc13mutG4mut-1.4 orientation C*	This study
