# Supplementary material for: Stimulation of Gross Chromosomal Rearrangements by the Human CEB1 and CEB25 Minisatellites in Saccharomyces cerevisiae Depends on G-Quadruplexes or Cdc13
Source: PLoS Genet. 2012 Nov 1;8(11):e1003033. doi: 10.1371/journal.pgen.1003033 (PMC3486850; doi:10.1371/journal.pgen.1003033)
Supplement: Table S2 — Minisatellites used in this study. (PDF) [file pgen.1003033.s010.pdf]

Table S2

Name	Sequence (5'- 3')	Nb. of mutations	TG/GG/GT density G strand	TG/GG/GT density C strand	GC content (%)
CEB1-WT	GGGGGGAGGGAGGGTGGCCTGCGGAGGTCCCTGGGCTGA	NA	0,52	0,08	77
CEB1-Gmut	GCGCGGAGTGAGAGTGGCCTGCGGAGGTCCCTGCGCTGA	5	0,31	0,08	72
CEB25-WT	AAGGGTGGGTGTAAGTGTGGGTGGGTGTGAGTGTGGGTGTGGAGGTAGATGT	NA	0,75	0,00	56
CEB25-Cdc13mut	CAGGGCGGGTCTAAGTTAGGGCGGGATTTAGTCAGGGACTCGAGGATGATGT	16	0,33	0,06	56
CEB25-Cdc13mut-Gmut	CAGCGCGCGTCTAAGTTAGCGCGCGATTTAGTCAGGGACTCGAGGATGATGT	20	0,18	0,06	56
hRAS1	CCCCTGGAGAGAAGGGGGAGTGTGGCGT	NA	0,44	0,11	68
