# Supplementary material for: Stimulation of Gross Chromosomal Rearrangements by the Human CEB1 and CEB25 Minisatellites in Saccharomyces cerevisiae Depends on G-Quadruplexes or Cdc13
Source: PLoS Genet. 2012 Nov 1;8(11):e1003033. doi: 10.1371/journal.pgen.1003033 (PMC3486850; doi:10.1371/journal.pgen.1003033)
Supplement: Table S3 — GCR rates measured in the untreated cells (untreated sheet) and in cells treated with G-quadruplex ligands (Phen-DC sheet). The upper and lower 95% confidence intervals, as well as the number of independent cultures performed (n) are indicated. The number after the minisatellite name (CEB1-WT-) indicates its size in kb. The number of motifs has been determined by sequencing of the array for CEB1-Gmut-1.7 [16], HRAS1-0.7, CEB25-WT-0.7, CEB25-Cdc13mut-1.4 and CEB25-Cdc13mut-Gmut-1.4, and estimated based on their size for the various CEB1-WT and CEB1-Gmut alleles. (PDF) [file pgen.1003033.s011.pdf]

Table S3 (Untreated)

Strain	Minisatellite	Number of motif	Orientation	Genotype	GCR rate	95% IC up	95% IC low	n
ORT6531	None			WT	4.30E-10	4.63E-09	4.30E-10	11
ORT6568	None			*pif1Δ*	1.01E-06	1.56E-07	4.42E-07	20
ORT6542-8	CEB1-WT-0.66	17	G	WT	1.10E-08	9.95E-09	2.75E-09	25
ORT6542-5	CEB1-WT-1.2	31	G	WT	3.86E-07	6.93E-07	1.93E-07	15
ORT6542-6	CEB1-WT-1.7	43	G	WT	6.97E-07	8.87E-08	1.05E-07	45
ORT6542-4	CEB1-WT-1.9	49	G	WT	1.63E-06	1.72E-06	4.25E-07	10
ORT6542-1	CEB1-WT-2.7	70	G	WT	1.59E-05	7.92E-06	7.33E-07	15
ORT6591-4	CEB1-WT-0.45	11	C	WT	1.57E-08	9.03E-09	6.44E-09	10
ORT6591-3	CEB1-WT-0.8	20	C	WT	3.25E-07	1.15E-07	2.12E-07	10
ORT6591-8	CEB1-WT-1.1	28	C	WT	3.45E-07	3.83E-07	1.63E-07	10
ORT6591-1	CEB1-WT-1.7	43	C	WT	7.48E-07	1.10E-07	1.50E-07	25
ORT6543-10	CEB1-WT-0.2	5	G	*pif1Δ*	2.99E-06	5.18E-07	1.72E-06	15
ORT7154-2	CEB1-WT-0.3	8	G	*pif1Δ*	3.45E-06	9.93E-07	1.31E-06	10
ORT7154-7	CEB1-WT-0.7	18	G	*pif1Δ*	7.54E-06	7.62E-06	7.54E-06	15
ORT7154-3	CEB1-WT-0.9	23	G	*pif1Δ*	1.93E-05	3.70E-06	5.28E-06	15
ORT6543-4	CEB1-WT-1.5	38	G	*pif1Δ*	7.31E-05	1.67E-05	5.06E-05	10
ORT6543-1	CEB1-WT-1.7	43	G	*pif1Δ*	3.89E-04	6.05E-05	8.67E-05	16
ORT6541-1	CEB1-WT-0.15	4	C	*pif1Δ*	3.18E-06	8.89E-07	6.28E-07	10
ORT6592-1	CEB1-WT-0.36	9	C	*pif1Δ*	4.57E-06	7.46E-07	1.06E-06	10
ORT6592-4	CEB1-WT-0.9	23	C	*pif1Δ*	1.14E-05	5.12E-06	5.57E-06	10
ORT6592-18	CEB1-WT-1.2	31	C	*pif1Δ*	2.39E-05	5.10E-06	2.11E-06	10
ORT6592-22	CEB1-WT-1.4	36	C	*pif1Δ*	2.53E-04	6.22E-05	1.03E-04	12
ORT7153-9	CEB1-WT-1.7	43	C	*pif1Δ*	4.60E-04	7.05E-05	8.10E-05	14
ORT7189-1	CEB1-WT-1.7	43	G	*rad51Δ*	2.92E-07	1.04E-07	1.08E-07	11
ORT7191-3	CEB1-WT-1.7	43	C	*rad51Δ*	1.77E-07	1.48E-07	1.77E-07	11
ORT7310-2	CEB1-WT-1.7	43	G	*rad52Δ*	3.55E-07	5.10E-08	1.01E-07	11
ORT7312-5	CEB1-WT-1.7	43	C	*rad52Δ*	1.68E-07	8.09E-08	2.13E-08	10
ORT7309-3	CEB1-WT-1.7	43	C	*dnl4Δ*	5.26E-07	2.00E-07	1.77E-07	11
ORT7188-3	CEB1-Gmut-0.9	23	G	WT	8.64E-09	4.16E-09	2.46E-09	11
ORT6550-2	CEB1-Gmut-1.7	42	G	WT	2.77E-08	8.76E-09	9.95E-09	40
ORT6550-7	CEB1-Gmut-1.9	49	G	WT	3.52E-08	6.42E-09	2.68E-08	10
ORT7188-2	CEB1-Gmut-2.2	56	G	WT	1.39E-07	4.99E-08	3.67E-08	11
ORT6548	CEB1-Gmut-1.7	42	C	WT	2.07E-08	5.90E-09	3.53E-09	20
ORT6551-5	CEB1-Gmut-0.9	23	G	*pif1Δ*	2.30E-06	4.78E-07	8.90E-07	10
ORT6551-4	CEB1-Gmut-1.3	33	G	*pif1Δ*	3.25E-06	1.67E-06	1.19E-06	10
ORT6551-1	CEB1-Gmut-1.7	42	G	*pif1Δ*	6.32E-06	1.98E-06	8.41E-07	15
ORT6549	CEB1-Gmut-1.7	42	C	*pif1Δ*	3.05E-06	7.34E-07	1.27E-06	20
ORT7182	hRAS1-0.7	26	G	WT	8.48E-09	1.16E-08	2.55E-09	11
ORT7183	hRAS1-0.7	26	C	WT	1.11E-08	5.91E-09	1.11E-08	10
ORT7322	hRAS1-0.7	26	G	*pif1Δ*	3.68E-06	9.56E-07	1.07E-06	15
ORT7323	hRAS1-0.7	26	C	*pif1Δ*	3.21E-06	4.27E-07	1.62E-06	9
ORT6558-1	CEB25-WT-0.7	13	G	WT	1.16E-04	2.55E-05	2.78E-05	15
ORT6556-1	CEB25-WT-0.7	13	C	WT	2.24E-07	4.84E-08	9.53E-09	10
ORT6559-5	CEB25-WT-0.7	13	G	*pif1Δ*	1.22E-04	1.04E-04	6.01E-05	15
ORT6557-1	CEB25-WT-0.7	13	C	*pif1Δ*	2.42E-06	1.16E-06	6.44E-07	15
ORT7344	CEB25-WT-0.7	13	G	*rad52Δ*	3,94E-04	1,40E-04	3,58E-04	10
ANT1181-1	CEB25-Cdc13mut-1.4	27	G	WT	3.07E-07	9.13E-08	6.43E-08	20
ANT1180-5	CEB25-Cdc13mut-1.4	27	C	WT	2.95E-07	1.33E-07	5.26E-08	18
ANT1185-4	CEB25-Cdc13mut-1.4	27	G	*pif1Δ*	3.86E-06	1.08E-06	4.32E-07	20
ANT1184-1	CEB25-Cdc13mut-1.4	27	C	*pif1Δ*	2.89E-06	1.59E-06	2.74E-07	19
ANT1183-1	CEB25-Cdc13mut-Gmut-1.4	27	G	WT	3.55E-07	1.09E-07	1.40E-07	20
ANT1182-1	CEB25-Cdc13mut-Gmut-1.4	27	C	WT	2.37E-07	1.32E-07	5.84E-08	18
ANT1187-1	CEB25-Cdc13mut-Gmut-1.4	27	G	*pif1Δ*	2.52E-06	1.84E-06	3.77E-07	18
ANT1186-1	CEB25-Cdc13mut-Gmut-1.4	27	C	*pif1Δ*	2.07E-06	6.45E-07	3.70E-07	25

Table S3 (Phen-DC-treated)

Strain	Minisatellite	Number of motif	Orientation	Genotype	Treatment	GCR rate	95% IC up	95% IC low	n	Fold vs. untreated	Fold vs. WT
ORT6531	None			WT	Phen-DC_3 10 µM	7.18E-09	3.33E-09	2.50E-09	11	17	NA
ORT6542-6	CEB1-WT-1.7	43	G	WT	Phen-DC_3 1 µM	3.29E-06	1.39E-06	1.04E-06	10	5	NA
ORT6542-6	CEB1-WT-1.7	43	G	WT	Phen-DC_3 5 µM	1.14E-05	5.95E-07	1.06E-06	10	16	NA
ORT6542-6	CEB1-WT-1.7	43	G	WT	Phen-DC_3 10 µM	3.66E-05	1.00E-05	3.44E-06	25	52	NA
ORT6542-6	CEB1-WT-1.7	43	G	WT	Phen-DC_6 1 µM	5.11E-06	1.82E-06	2.68E-06	10	7	NA
ORT6542-6	CEB1-WT-1.7	43	G	WT	Phen-DC_6 5 µM	1.19E-05	1.93E-06	2.63E-06	10	17	NA
ORT6591-1	CEB1-WT-1.7	43	C	WT	Phen-DC_3 10 µM	1.66E-05	4.99E-06	6.89E-06	20	22	NA
ORT6550-2	CEB1-Gmut-1.7	42	G	WT	Phen-DC_3 10 µM	3.57E-08	2.24E-08	7.39E-09	25	1.3	NA
ORT6550-2	CEB1-Gmut-1.7	42	G	WT	Phen-DC_6 5 µM	1.38E-08	1.71E-08	1.38E-08	10	0.5	NA
ORT6548	CEB1-Gmut-1.7	42	C	WT	Phen-DC_3 10 µM	1.59E-08	6.34E-09	5.51E-09	22	0.8	NA
ORT6556-1	CEB25-WT-0.7	13	C	WT	Phen-DC_3 10 µM	2.00E-07	3.56E-08	7.28E-08	10	0.9	NA
ORT6557-1	CEB25-WT-0.7	13	C	*pif1Δ*	Phen-DC_3 10 µM	1.34E-05	5.56E-06	3.26E-06	10	5.5	NA
ANT1181-1	CEB25-Cdc13mut-1.4	27	G	WT	Phen-DC_3 10 µM	3.25E-07	9.95E-08	1.09E-07	10	1.1	NA
ANT1180-5	CEB25-Cdc13mut-1.4	27	C	WT	Phen-DC_3 10 µM	3.22E-07	4.11E-07	6.60E-08	10	1.1	NA
ANT1185-4	CEB25-Cdc13mut-1.4	27	G	*pif1Δ*	Phen-DC_3 10 µM	9.00E-06	4.40E-06	1.49E-06	10	2.3	NA
ANT1184-1	CEB25-Cdc13mut-1.4	27	C	*pif1Δ*	Phen-DC_3 10 µM	1.32E-05	8.60E-06	1.40E-06	10	4.6	NA
ANT1183-1	CEB25-Cdc13mut-Gmut-1.4	27	G	WT	Phen-DC_3 10 µM	1.97E-07	6.39E-08	7.26E-08	10	0.6	NA
ANT1182-1	CEB25-Cdc13mut-Gmut-1.4	27	C	WT	Phen-DC_3 10 µM	2.04E-07	1.00E-07	3.93E-08	10	0.9	NA
ANT1187-1	CEB25-Cdc13mut-Gmut-1.4	27	G	*pif1Δ*	Phen-DC_3 10 µM	1.82E-06	2.55E-06	7.43E-07	10	0.7	NA
ANT1186-1	CEB25-Cdc13mut-Gmut-1.4	27	C	*pif1Δ*	Phen-DC_3 10 µM	8.45E-07	8.16E-07	2.96E-07	13	0.4	NA
ORT7189-1	CEB1-WT-1.7	43	G	*rad51Δ*	Phen-DC_3 10 µM	1.13E-05	5.92E-06	7.61E-07	16	39	0.31
ORT7191-3	CEB1-WT-1.7	43	C	*rad51Δ*	Phen-DC_3 10 µM	5.27E-06	4.23E-06	1.71E-06	21	30	0.14
ORT7310-2	CEB1-WT-1.7	43	G	*rad52Δ*	Phen-DC_3 10 µM	7.12E-06	6.45E-06	7.12E-06	10	20	0.19
ORT7312-5	CEB1-WT-1.7	43	C	*rad52Δ*	Phen-DC_3 10 µM	1.48E-06	6.31E-07	2.25E-07	6	9	0.04
