# Supplementary material for: Stimulation of Gross Chromosomal Rearrangements by the Human CEB1 and CEB25 Minisatellites in Saccharomyces cerevisiae Depends on G-Quadruplexes or Cdc13
Source: PLoS Genet. 2012 Nov 1;8(11):e1003033. doi: 10.1371/journal.pgen.1003033 (PMC3486850; doi:10.1371/journal.pgen.1003033)
Supplement: Table S6 — Primers used in this study. Sequences are oriented 5′ to 3′. (PDF) [file pgen.1003033.s014.pdf]

Table S6

Primer	Sequence (5' - 3')
RAD51A	TTGAGCATTCCCTGAGCATT
RAD51B	TCCCCTAAAAGGATAAAGCCG
RAD52A	GTGAAATCACCACAGTTTGGA
RAD52B	ACCTAAGGATTCCGCTGAAA
DNL4A	GGGTAATGGTTCGCAATCTG
DNL4B	GCGGTGCAAATAAACACAGG
