# Supplementary material for: Stimulation of Gross Chromosomal Rearrangements by the Human CEB1 and CEB25 Minisatellites in Saccharomyces cerevisiae Depends on G-Quadruplexes or Cdc13
Source: PLoS Genet. 2012 Nov 1;8(11):e1003033. doi: 10.1371/journal.pgen.1003033 (PMC3486850; doi:10.1371/journal.pgen.1003033)
Supplement: Text S1 — Supplementary Methods. (DOCX) [file pgen.1003033.s015.docx]

**Supplemental data**

**Supplementary Methods**

**Pulse-field gel electrophoresis.**

The sizes of rearranged chromosomes V were determined by pulsed-field gel electrophoresis analysis and Southern blotting techniques. Yeast chromosomal DNA blocks were prepared by mixing 1.25 ml of yeast cells from stationary-phase cultures with 1 % low melting point agarose (Cambrex) as described previously [[1](#_ENREF_1)]. PFGE was performed with the CHEF-DR III system (Bio-Rad). Chromosomes were separated on a 1 % agarose gel in 0.5 X Tris-borate-EDTA (TBE) buffer at 14°C for 24 h at 6.0 V/cm (120 V) with a 120°C included angle and a 60- to 120-s linear switch time ramp. DNA was transferred onto a Hybond-XL membrane (Amersham Biosciences), and probed with a αdCTP^32^-radiolabeled PCR fragment of 1759 bp corresponding to the *FCY2* gene (chV:266,200-267,959).

**Comparative Genomic Hybridization**

RDKY3615, ORT6591-1 and 4 Can/5FOA-resistant clones were analyzed by comparative genomic hybridization microarrays (CGH). Yeast cells grown in 5 ml YPD medium to the stationary phase were harvested and the pellet resuspended in 500 µl sorbitol solution (1 M sorbitol, 0.1 M EDTA pH 8.0 and 0.3 mg/ml Zymolyase 20-T). The sample solution was incubated at 37°C for 1 hour. Cells were then resuspended in 500 µl Tris-HCl buffer (50 mM Tris-HCl pH 8.0, 50 mM EDTA and 100 mM NaCl) and genomic DNA was extracted with phenol/chloroform/isoamyl alcohol (25:24:1). After isopropanol precipitation, the pelleted genomic DNA was dissolved in 300 µl TE buffer. To denature RNA, 1 µl DNase-free RNase A (30 mg/ml) was added to the sample solution and incubated at 37°C for 30 min. After RNase treatment ethanol precipitation was performed. Pelleted genomic DNA was dissolved in 100 µl TE buffer.

For each labeling, 120 ng genomic DNA sample was digested by *Hinc*II and *Ssp*I (0.5 unit) to an average length of 0.5 to 2 kb. After purification with a Nucleospin Extract II kit (Macherey-Nagel) the sheared genomic DNA was random-primer labeled using a BioPrime DNA labeling system (Invitrogen), to include in a 50 µl reaction, dATP, dGTP, and dCTP (250 µM each), dTTP (110 µM) and amino-allyl dUTP (150 µM). The labeled products were purified and concentrated using a Microcon YM-30 filter (Amicon Millipore). Cy3 and Cy5 (Amersham, GE) were then separately coupled to the appropriate DNA (4 µg) with 50 mM Na_2_CO_3_ pH 9.0 for 1 hour in the dark. The appropriate Cy3- and Cy5-labelled samples were pooled and purified using the Nucleospin Extract II kit (Macherey-Nagel). DNA samples were hybridized on an Agilent 44k yeast whole genome oligonucleotide array for 16h at 65°C in the 1 x hybridization buffer supplied by Agilent. Slides were washed as described in [[2](#_ENREF_2)]. Microarray images were acquired using a GenePix 4000 scanner and data were quantified using GenePix Pro 5.1 software (Axon Instruments). For all microarray experiments, the median local background intensity was subtracted from the median spot intensity before calculating for each spot the intensity ratio (Cy5/Cy3 median intensity). The log2 of this ratio is plotted in Figure S5B.

**Sequencing of *CAN1* and *URA3***

*CAN1* has been PCR amplified with primers CAN1extF2 and CAN1extR2 and sequenced using these primers and CAN1intF2, CAN1intF3, CAN1intR2 and CAN1intR3 [[3](#_ENREF_3)]. *URA3* has been PCR amplified with primers URA3extF and URA3extR, and sequenced using these primers and URA3intF2 and URA3intR2 [[3](#_ENREF_3)]. Sequences were analyzed with SeqScape 2.5 (Applied Biosystems).

**Supplementary References**

1. Murakami H, Borde V, Nicolas A, Keeney S (2009) Gel electrophoresis assays for analyzing DNA double-strand breaks in Saccharomyces cerevisiae at various spatial resolutions. Methods Mol Biol 557: 117-142.

2. Buhler C, Borde V, Lichten M (2007) Mapping meiotic single-strand DNA reveals a new landscape of DNA double-strand breaks in Saccharomyces cerevisiae. PLoS Biol 5: e324.

3. Lang GI, Murray AW (2008) Estimating the per-base-pair mutation rate in the yeast Saccharomyces cerevisiae. Genetics 178: 67-82.
